# Carbon nanomaterials-Based Inks and Electrodes Using Chitin Nanocrystals

**DOI:** 10.1021/acssuschemeng.4c05253

**Published:** 2024-10-14

**Authors:** Víctor Calvo, Carlos Martínez-Barón, Benjamín Vázquez-Conejo, Antonio Dominguez-Alfaro, Antonio J. Paleo, Belén Villacampa, Alejandro Ansón-Casaos, Wolfgang K. Maser, Ana M. Benito, José M. González-Domínguez

**Affiliations:** † 16379 Instituto de Carboquímica ICB-CSIC, C/Miguel Luesma Castán 4, 50018 Zaragoza, Spain; ‡ Faculty of Chemistry, University of Basque Country, Paseo Manuel Lardizabal 3, 20018 Donostia-San Sebastián, Spain; § 2C2T-Centre for Textile Science and Technology, University of Minho, Campus de Azurém, 4800-058 Guimarães, Portugal; ∥ Instituto de Nanociencia y Material (INMA), 16765 CSIC-Universidad de Zaragoza, 50009 Zaragoza, Spain

**Keywords:** biopolymers, chitin nanocrystals, carbon nanomaterials, conductive inks, electrodes

## Abstract

Dispersing 1D carbon nanomaterials (CNMs) for film processing traditionally relies on surfactants or organic solvents. These methods, however, raise environmental concerns and can negatively impact the final properties of the CNMs. In this work we demonstrate that chitin nanocrystals (ChNCs) synthesized via acid hydrolysis provide a greener pathway for the development of waterborne CNMs-based inks, including single-walled carbon nanotubes, multiwalled carbon nanotubes, and carbon nanofibers. Various concentrations of ChNCs were mixed with each type of CNM to maximize the CNM concentration within the ink. In-depth characterization of the CNM/ChNC inks preceded their processing into supported conductive films by spray-coating. A subsequent thermal treatment of ChNCs at 450 °C leads to the efficient removal of ChNC and results in a significant enhancement of the electrical conductivity. Moreover, freeing the CNMs network structure contributes to increased electrochemical performance of the treated films, as expressed by improved Faradaic charge transfer efficiencies and kinetics reflected by 1 order of magnitude reduced rate constants, when tested with various redox probes. Our findings highlight the potential of ChNCs as a sustainable processing adjuvant of CNMs, leading to electrically conductive electrodes with suitable electrochemical properties for their use in diverse devices and applications.

## Graphical abstract

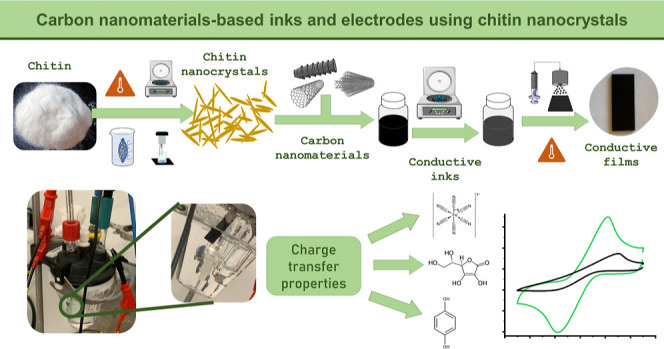

## Introduction

Carbon nanomaterials (CNMs), such as graphene derivatives or carbon nanotubes, exhibit excellent electronic and mechanical properties, which makes them suitable for a wide range of applications.^1–3^ A powerful approach for translating CNM properties from the nanoscale to the microscale is by assembling films from these materials.^4–6^ Such films could offer high performance and cost-effectiveness, becoming essential components in a variety of devices, ranging from sensors^7^ to transparent electrodes in solar cells,^8^ or energy storage device components.^9,10^ However, the conventional use of surfactants and organic solvents to disperse them negatively affects the CNM properties and the environmental compatibility of these devices.^11^ These surfactants can compromise the electrical properties of CNMs by altering their intrinsic characteristics and reducing their exposed surface area, thereby limiting their effectiveness in their target applications. In the search for environmentally friendlier alternatives, the use of nanostructured biopolymers to achieve water-based CNM dispersions emerges as a promising solution for the development of sustainable devices based on CNMs.^6,12,13^

Chitin, a naturally abundant biopolymer, can be extracted from various sources, such as the exoskeletons of various animals, namely, shrimps and silkworms.^14–16^ However, despite its versatility in diverse applications (e.g., fertilizers, surgical sutures, and tissue engineering materials),^14,15^ its potential remains largely underexploited. Chitin, known for its excellent biocompatibility, biodegradability, and high mechanical strength, is composed of repeating units of β-(1–4)-*N*-acetylglucosamine.^16^ Traditionally, efforts to revalorize chitin involve deacetylating it to produce chitosan, a water-soluble material. However, chitosan has several drawbacks: apart from inferior physical properties, the requirement for acidic conditions for dissolution increases production costs and limits its sustainable footprint.^14–16^ Overcoming these issues, chitin nanocrystals (ChNCs), also known as chitin nanowhiskers, emerge as a promising alternative to chitosan. ChNCs can be prepared from bulk chitin through either chemical or physical methods, exhibiting improved properties and enhanced water stability with respect to bulk chitin.^17,18^ Several methods exists for the isolation of ChNCs,^17,18^ whereby acid hydrolysis, known for its efficiency, is a common choice. It is often combined with ultrasound treatment or mechanical disintegration to improve ChNCs’ dispersibility.^16–19^ Among the various methods for synthesizing ChNCs, acid hydrolysis with HCl is a relatively greener approach compared with other recurrent methods that rely on ionic liquids, harsh oxidative treatments, or chemical deacetylation processes. This is also justified by the nonvolatile nature of the herein employed HCl concentrations, which entails significantly lower environmental impacts on air and soil pollution as well as the wide accessibility of HCl. The unique properties of ChNCs, including their liquid crystal behavior, antibacterial and antioxidant properties, and thermal insulation capabilities, broaden the applications of chitin-derived materials.^15,20^ These materials find uses in diverse fields, such as cosmetics, health care, material science, and food processing. ChNCs demonstrate versatility and potential to address new needs, for instance in stabilizing oil-in-water emulsions^21,22^ and act as nanofillers in biocomposites.^19,23^ Impressively, ChNCs improve the mechanical properties of nanocomposites,^14,19,24^ highlighting the superior strength of ChNCs compared to that of its closest analogue, cellulose nanocrystals.

Although several studies have successfully used cellulose nanocrystals for dispersing single-walled carbon nanotubes (SWCNTs), multiwalled carbon nanotubes (MWCNTs), and carbon nanofibers (CNFs) in water,^3,6,12,13,25^ the use of ChNCs remains largely underexplored for this purpose. One key difference between ChNCs and cellulose nanocrystals lies in their surface charge. Hydrochloric acid treatment gives ChNCs an electropositive surface charge due to the protonation of residual amino groups,^21,26^ in contrast to the negatively charged sulfate ester groups in cellulose nanocrystals.^27^ While a recent research study has demonstrated the potential of ChNCs in dispersing MWCNTs in water,^28^ further research is needed. This includes optimizing the amount of ChNCs needed for effective dispersion, investigating other one-dimensional CNMs, conducting in-depth characterization to gain a deeper understanding of the interaction between ChNCs and CNMs, and exploring alternative processing methods. This approach will unveil the full potential of using ChNCs and their benefits for dispersing CNMs in a variety of applications.

The present work (Scheme 1) explores a greener approach to disperse hydrophobic CNMs (SWCNTs, MWCNTs, and CNFs) in water, taking advantage of ChNCs, synthesized via acid hydrolysis. These dispersions were employed as inks in the fabrication of carbon-based films on glass substrates through spray coating, a processing method that allows high control over the final film properties and is ready to scale-up. The surface resistivity of the resulting films improved after thermal treatment under a N_2_ atmosphere. Subsequently, electrochemical characterization was carried out to gain more insights into the effect of the ChNCs pyrolysis toward their charge transfer properties. Our strategy allows the fabrication of conductive films with good electrical and electrochemical properties. Despite the intrinsic insulating nature of this nanostructured biopolymer, ChNCs are capable of serving as functional components in devices, thus expanding their potential applications.

**Scheme 1:**
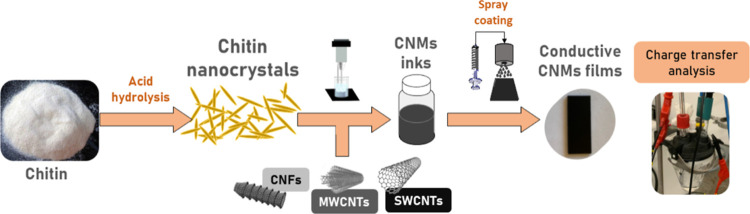
General Scheme of the Work Herein Reported

## Experimental Section

### Materials and Reagents

Chitin extracted from shrimp shells (practical grade, powder; C7170) was purchased from Sigma-Aldrich and hydrochloric acid 37% (AGR IS; CHAC-0AI) was purchased from Labkem (Spain). Ultrapure water, purified with a Siemens Ultraclear device, was used throughout all of the experimental procedures. SWCNTs were acquired from Carbon Solutions Inc. (AP-SWNT and P2-SWNT grades, Riverside, CA, USA).^29^ P2-SWCNTs, as reported by the manufacturer, have over 90% carbonaceous purity and between 4% and 8% metal content. MWCNTs were purchased from Nanocyl (NC 7000, Sambreville, Belgium).^30^ These MWCNTs were produced via Catalytic Chemical Vapor Deposition and have a 90% of carbon purity, an average length of 1.5 μm, and an average diameter of 9.5 nm. CNFs (Pyrograf III PR 24 LHTXT, ASI, Cedarville, OH, USA) were produced by catalytic chemical vapor deposition in a horizontal reactor at 1100 °C followed by a thermal post-treatment at 1500 °C under an inert atmosphere. The CNFs have an average diameter of around 100 nm with a dual-wall structure surrounding a hollow tubular core with a diameter of around 20 nm, and lengths from 50 to 100 μm.^31^

### ChNCs Synthesis

The presented synthesis was modified from a previously optimized protocol by Narkevicius et al.,^26^ using a final centrifugation to ensure that chunks and nondispersed particles were removed. The first step was the preparation of an 80 mL 3 M HCl solution and its subsequent heating to 100 °C. After that, 4 g of chitin powder was added, and the mixture was refluxed for 90 min at 110–120 °C, keeping the flask connected to a condenser tube with tap water flowing within at 20 °C to ensure that the volume remains constant. Subsequently, the hydrolyzed medium was poured into 1 L of ultrapure water at 4 °C. It was then stored overnight in the fridge at 4 °C, and the supernatant liquid was decanted off. The sediment was inserted into a dialysis sack (D9652, Merck, regenerated cellulose, with an average flat width of 33 mm) and then dialyzed against ultrapure water in a 5 L beaker. The dialysis water was replaced every 3–4 h until no acid was detected. The following step involved a mild sonication using an ultrasonic tip (Hielscher DRH-P400S; 400 W maximum power; 24 kHz maximum frequency at 20% amplitude, and 50% cycle time) for 20 min. Subsequently, the liquid was subjected to centrifugation at 9000 rpm (9327 rcf) for 20 min, keeping the supernatant fraction while the solid pellet was redispersed using fresh ultrapure water and centrifuged under identical conditions. This process was repeated twice, ensuring that no more ChNCs were extracted from the pellets. The as-obtained ChNCs aqueous colloids were characterized by dynamic light scattering (DLS) and ζ-potential analysis. Besides, three aliquots of 30 mL were freeze-dried and weighed to measure the concentration and the mass yield. The resulting solid nanocrystals were characterized by powder X-ray diffraction (XRD), thermogravimetric analysis (TGA), and Fourier transform infrared spectroscopy (FTIR).

### Pretreatment of CNMs

Prior to the application of CNMs, surface conditioning was carried out to improve their hydrophilicity. AP-SWNT were purified by the supplier resulting in P2-SWNT, and the latter was used as received. MWCNTs were modified by a mild oxidation process using diluted HNO_3_ under reflux, a typically employed method for purifying carbon nanotubes.^32,33^ Briefly, commercial MWCNTs were added to a 1.5 M HNO_3_ solution and refluxed for 90 min. Then, the resulting mixture was filtered through Isopore membrane filters (10 μm TCTP) and dried overnight at 65 °C. The as-received CNFs were modified using H_2_O_2_.^34^ CNFs were mixed with aqueous 1.5 M H_2_O_2_ and kept under reflux conditions for 90 min. Subsequently, the mixture was filtered through Isopore Membrane Filters (10 μm TCTP) and dried overnight at 65 °C.

### Characterization of CNMs

To gain insight into surface modification, the CNMs were characterized by four techniques: XRD, Raman spectroscopy, TGA, and X-ray photoelectron spectroscopy (XPS). Note that the as-received CNMs (AP-SWNTs, NC 7000 and Pyrograf III) were used as reference for morphological and structural characterization of the employed CNMs. See Supporting Information for full details and data (Figures S1 and S2 and Table S1).

### Preparation and Optimization of CNM/ChNC Inks

The concentration of ChNCs (1–5 g/L) was optimized, maintaining an initial concentration of 1 g/L of each purified CNM (SWCNTs, MWCNTs, or CNFs). CNMs and ChNCs were first mixed in solid form and then incorporated into 20 mL of ultrapure water. The dispersion process was conducted with an ultrasound tip (Hielscher DRH-P400S) at a 60% amplitude and 0.5 cycles, keeping the dispersion in an ice/water bath to reduce its heating, along with manual shaking and bath sonication. This method induces the interaction between both materials, resulting in the formation of stable suspensions.^12,25^ To separate nonstabilized particles and to eventually obtain aqueous inks, the suspensions were centrifuged at 4000 rpm (1842 rcf) for 4 min. Subsequently, these CNM inks were diluted and analyzed by DLS and ζ-potential at a pH of 4.6. Moreover, these diluted samples were characterized by UV–vis spectroscopy in a Shimadzu UV-2401PC spectrophotometer to estimate the concentration of CNMs based on their corresponding absorbance ratios. Absorbance of the SWCNTs and MWCNTs inks were recorded at 850 nm, whereas 650 nm was selected for CNFs. The Supporting Information section contains calculations of the CNMs’ concentrations (eq S1). Each optimal ink was lyophilized, and the solids were characterized by XRD, TGA under a N_2_ atmosphere, and Raman spectroscopy before and after a thermal treatment at 450 °C for 30 min under a N_2_ atmosphere.

### Preparation of CNM/ChNC Films

Before the deposition of the CNM/ChNC films, the glass substrates (1 cm × 2.5 cm) were cleaned by subsequent immersion in acetone and isopropanol for 15 min each with an ultrasonic bath, being immediately dried with a N_2_ stream. Then, the glass substrates were ozonized (Ossila UV Ozone Cleaner) for 15 min, ensuring an adequate adhesion of the film. The CNM/ChNC inks were deposited using a semiautomatic spray coater (Nadetech ND-SP Ultrasonic PRO) at a flow rate of 42 mL/h. The nozzle was placed 60 mm above the sample, and the temperature of the hot plate was set at 80 °C. Under these conditions, we were able to control the film thickness by adjusting the number of spray coating steps, which approximately requires 12 s each. Additionally, the resulting electrodes were thermally treated in a horizontal reactor at 450 °C for 30 min under a N_2_ atmosphere.

### Characterization of CNM/ChNC Films

The sheet resistance (*R*_S_) of the conductive films over glass was measured with an in-line 4-point probe configuration in a Keithley 4200 semiconductor characterization system with equidistant probe separations of 2.24 mm.^35^ Surface morphology of films was evaluated by scanning electron microscopy (SEM) images, which were acquired directly, with no preparation procedure, before and after the thermal treatment with a JEOL JSM 6400 microscope at 20 kV of voltage and a working distance of 8 mm without metallization. XPS was used to compare the surface atomic composition of the CNM/ChNC films before and after thermal treatment. Measurements were carried out using an ESCA+ (Omicron) equipment, provided with a dual X-ray source (Mg/Al), a hemispheric analyzer, and a 7-channeltron detector, working under high vacuum conditions (2 × 10^–7^ mbar). A monochromatic radiation of 1486.68 eV (Al Kα line) was used to irradiate samples perpendicularly at 12 kV. The register settings were established as a 50 ms accumulation time for each step in all cases. Survey spectra were recorded by 1 eV energy steps and 20 scans, while 0.1 eV steps were taken for specific regions (30 scans in C 1s and 40 scans in O 1s and N 1s). The obtained spectra were calibrated in the C 1s region by taking the C=C contribution as 284.4 eV.^36^ The numerical treatment was performed with the CASA-XPS software tool, and the background corrections were effected with Shirley functions. The deconvoluted spectra for each specific region (C 1s, O 1s, and N 1s) were obtained after imposing several constraints whenever necessary in order to maximize the quality of the fitting. These constraints were mainly those concerning the peak full width at half-maximum (fwhm), which was restricted to the 1.3–2.5 eV range, and the Gaussian-to-Lorentzian ratio, which was forced to be 70% in C 1s contributions, except for the C=C band at ∼284.4 eV, which additionally had symmetry corrections. For the rest of the components in O 1s and N 1s, a Gaussian–Lorentzian ratio of 30% was applied. An evaluation of the film thickness was conducted with a contact DektakXT Stylus Profiler (Bruker, Billerica, MA, USA), using a 2.5 μm radius stylus. In particular, the height was evaluated in different areas along the layer edge, thus obtaining the layer thickness as the mean value of such measurements. The contact angle of the films with ultrapure water were determined with an Attension Theta optical tensiometer (Biolin Scientific, Gothenburg, Sweden). Before the measurements, the films were placed on the tensiometer stage, and an ultrapure water droplet of approximately 0.2 μL was dispensed onto the film surface using a Hamilton syringe. The contact angles were then determined using OneAttension software by fitting a tangential line to the droplet’s interface with the film surface.

### Structural and Morphological Characterization

The ChNCs colloids and CNM/ChNC dispersions were characterized immediately after preparation by DLS and ζ-potential measurements in a nanosizer device (Malvern Nano ZS instrument). Particle size distribution was determined by irradiating the samples with a 633 nm He–Ne laser and applying the Stokes–Einstein equation. The ζ-potential was calculated on the basis of the electrophoretic mobility using Henry’s equation. For DLS measurements, a refractive index of 1.53 (chitin) was used for ChNCs, while a refractive index of 2.42 (carbon) was used for the CNM/ChNC dispersions. Each measurement was performed at least three times to ensure accuracy, with an average pH of 4.6 and an ambient temperature of 25 °C maintained throughout the experiments. XRD was conducted in a Bruker D8 ADVANCE diffractometer in a Bragg–Brentano geometry in the range 2θ = (5–40°), with steps of 0.05° and 3 s accumulation time. Viscosity measurements of the CNM/ChNC dispersions were performed in an Ubbelohde viscosimeter (type 0a). The correction of the kinematic energy (Hagenbach correction) was applied for adjusting the measurement to the employed viscosimeter. The kinematic viscosity (υ) was calculated as υ = *K*·*t*, where *K* is the constant of the viscosimeter, given as 0.009596 mm^2^/s^2^, and *t* is the time that the liquid needs to flow between the viscosimeter marks. The temperature was set at 30 °C and was controlled at ±0.01° with a Julabo thermostatic bath provided with an external temperature probe. The temperature calibration was performed by measuring the viscosity of ultrapure water. Each viscosity determination was repeated at least three times, achieving deviations of <1%, most typically around 0.1%. TGA was performed in a Netzsch TG 209F1 device, under a N_2_ or an air atmosphere, the heating rate being 10 °C/min, from room temperature to 800 °C. The FTIR spectrum was measured in a Bruker Vertex 70 spectrophotometer in the 400–4000 cm^–1^ range, mixing the ChNCs powder and the CNMs with spectroscopic-grade KBr. Raman spectroscopy was performed using a dispersive micro-Raman LabRam HR800 UV spectrometer (Horiba Jobin Yvon). The setup included a 532 nm excitation laser with an output power of 0.7 mW calibrated with a silicon standard, a CCD detector, and a confocal microscope equipped with a 100× objective lens. The sample was placed over a 600 g/mm diffraction grid of 600 g/mm. TEM was performed by using a Tecnai T20 (Thermofisher) equipped with a 200 kV field emission electron gun. Samples were prepared following the next protocol: the copper grid was placed on top of a sample droplet of 20 μL previously diluted to 0.1 g/L during 30 s, and the grid was dried under air conditions at room temperature for several hours.

### Electrochemical Characterization of CNM/ChNC Electrodes

Electrochemical measurements were carried out on a three-electrode configuration by using the Autolab PGSTAT 302N potentiostat (Metrohm AG, Herisau, Switzerland). The reference electrode was Ag/AgCl, 3 M NaCl (*E* ^0^ = 0.210 V), and the counter electrode was a graphite rod. The CNM/ChNC film was connected to the potentiostat probe through a copper wire and a contact made of silver conductive ink. Solutions of 0.1 M Na_2_SO_4_ (Sigma-Aldrich) and 0.1 M phosphate buffer (pH = 6.85, Sigma-Aldrich) were used as supporting electrolytes and were purged with N_2_ for 15 min prior to the cyclic voltammetry experiments. Faradaic transfer properties were evaluated by using three different redox probes: K_3_Fe­(CN)_6_/K_4_Fe­(CN)_6_ (Fisher Scientific, Madrid, Spain) in 0.1 M Na_2_SO_4_ and ascorbic acid and hydroquinone (Sigma-Aldrich) in 0.1 M phosphate buffer through cyclic voltammetry using a scan rate of 10 mV/s at room temperature (25 °C).

## Results and Discussion

ChNCs were successfully prepared and characterized using various techniques (Figure S3 and Table S2). Analysis revealed that freshly prepared ChNCs aqueous colloids show remarkable stability, underscored by a high positive ζ-potential value (45 ± 4 mV) and small mean hydrodynamic radius (115 ± 11 nm, with a polydispersity index of 0.39 ± 0.08) as determined by DLS analysis (Table S2). TEM images confirmed the typical needle-like morphology of ChNCs with a high aspect ratio, an average length of approximately 300–500 nm, and a diameter ranging from 20 to 40 nm (Figure S3D).

The CNMs have a low degree of functionalization (Figures S1 and S2 and Table S1), which is enough to adjust their surface hydrophilicity toward favorable interactions with ChNCs, without jeopardizing their structure and properties. However, this degree of functionalization is insufficient to allow for stable aqueous suspensions without the addition of dispersing agents. The optimization of CNM/ChNC ink formulations, driven by their colloidal properties, is crucial for achieving stable dispersions and functional films with desired characteristics. Considering the inherent electrically insulating properties of ChNCs, high concentrations of such nanostructured biopolymer within an ink formulation are likely to result in low electrical conductivity in the corresponding films. Hence, to maximize the film conductivity, the ChNC content needs to be optimized. Thereto, series of aqueous dispersions with initial ChNC concentrations ranging from 1.5 to 5 g/L were prepared while maintaining the initial CNM concentration at 1 g/L. After removing the nondispersed species through a centrifugation step, the colloidal properties and final CNM concentration of the inks were evaluated by UV–vis spectroscopy, DLS, and ζ-potential (Figure 1). The aim was to formulate inks with good colloidal stability, an adequate CNM concentration, and the minimum amount of ChNCs necessary for the preparation of conductive films. Figure 1 shows that the most stable dispersions with the highest concentration of CNMs were obtained with initial ChNC concentrations of 4 g/L for SWCNTs, 3.5 g/L for MWCNTs, and 3 g/L for CNFs. The relatively small hydrodynamic diameter of the aggregates, along with high ζ-potential values, confirms the good colloidal stability of these dispersions. It is important to note that the CNM/ChNC dispersions display positive ζ-potential values due to the prevalence of ChNCs, contrasting with the typical negative surface charge of oxidized 1D CNMs. For the SWCNTs-based ink, a ChNC concentration of 3 g/L was selected as the optimal due to the lower *R*_S_ value observed in the resulting film (Figure S4), underlining the significant impact of the nonconductive ChNCs on the electrical properties. Regarding MWCNTs, the maximum MWCNT concentration, close to 0.6 g/L, was achieved using 3.5 g/L ChNCs, which was significantly higher than that with 3.0 g/L ChNCs (Figure 1, central column). This MWCNT concentration was coupled to favorable colloidal parameters, including small aggregate sizes and ζ-potential values ranging between 20 and 25 mV, similar to those observed for the SWCNT inks. Investigation into CNFs revealed that at least 3 g/L ChNCs were required for their successful stabilization (Figure 1, right column), with ζ-potential values falling within the range of their nanotube analogues. To the best of our knowledge, these results represent the first report on the stabilization of CNFs in water using ChNCs and are among the pioneering studies using nanostructured biopolymers to stabilize CNFs,^12^ expanding the use of ChNCs as processing adjuvant of other CNMs. In summary, balancing the ChNC content with the desired CNM concentration is essential to ensure optimal colloidal stability and minimize the detrimental effects of ChNCs on the electrical properties. Our study broadens the application of ChNCs for dispersing CNMs in water, extending beyond MWCNTs to include SWCNTs and CNFs. We evaluated various ChNC concentrations using multiple techniques to ensure optimal dispersion.

**Figure 1:**
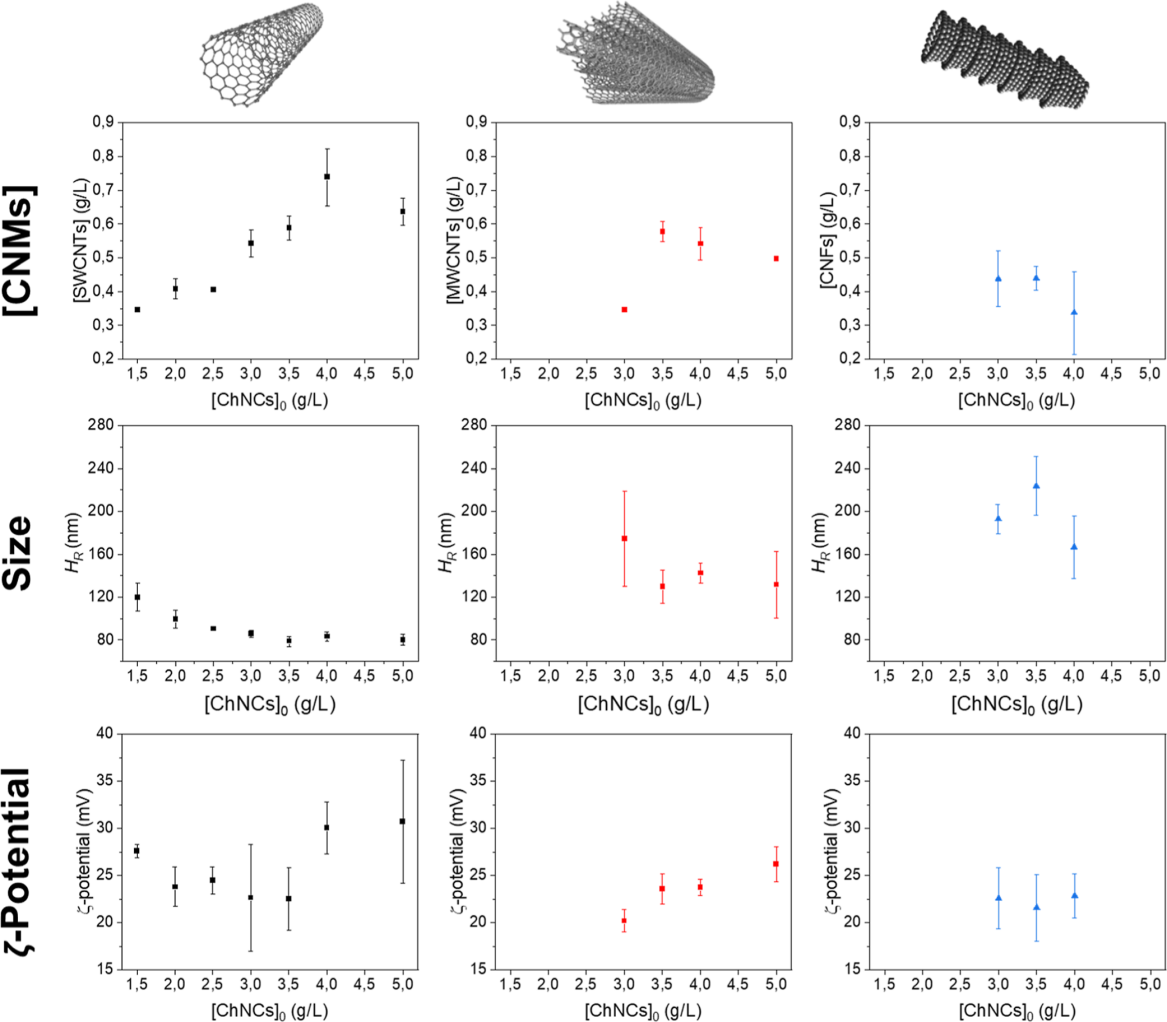
Colloidal properties of CNM-based aqueous inks with ChNCs. Each CNM is represented in different vertical columns: SWCNT/ChNC in black (left column), MWCNT/ChNC in red (central column), and CNF/ChNC in blue (right column). The *X* axis represents the initial ChNC concentration, while the final CNM concentration ([CNMs]), size, and ζ-potential are shown on the different *Y* axes. Note that in all cases, the starting CNM concentration was of 1 g/L. Data points where stable inks were not achieved across the entire concentration range (all below 3 g/L ChNC) have been omitted for clarity.

After selecting the favorable combinations of CNMs and ChNCs, the resulting dispersions (Figure S5) underwent characterization using various techniques to understand the interaction between the ChNCs and each CNM. UV–vis spectroscopy analysis of the samples (Figure S6) shows the characteristic profile of this type of CNMs, also featuring the metallic behavior of the SWCNTs (M_11_). TEM images of the SWCNT/ChNC aqueous dispersions clearly reveal how ChNCs needles discretely adsorb on the SWCNTs surface (Figure 2A,B). ChNCs cover most of their surface but are not completely parallel to the SWCNTs axis, resulting in a bramble-like morphology (Figure 2A,B). Additionally, Vis-NIR spectroscopy demonstrates the selective stabilization of nanotubes in dispersion compared with other carbon forms in the case of SWCNTs. This was ascertained using the NIR purity index calculated upon the *S*_22_ band according to the method by Itkis et al.,^37^ only applicable to SWCNT dispersions (see Figure S7 and Table S4 for the full data set). TEM images of MWCNT/ChNC (Figure 2C,D) and CNF/ChNC (Figure 2E,F) hybrids show how the ChNCs form a network around CNMs, heavily wrapping MWCNTs and CNFs in a notably different fashion from the SWCNTs case. Viscosity (υ) measurements at a constant temperature (30 °C) (Table S3) reveal values somewhat higher than that of ultrapure water (0.79 mm^2^/s) due to the presence of both CNMs and ChNCs. The υ of CNM inks with similar concentrations varied with their aspect ratio,^38^ with the SWCNTs ink showing the highest υ value (0.99 mm^2^/s) due to their superior aspect ratio compared to MWCNTs (0.91 mm^2^/s) and to CNFs (0.86 mm^2^/s). Despite these differences, the values remained close to those of water, highlighting the aqueous nature of the CNM/ChNC inks. Given the ζ-potential values, we initially hypothesized that electrostatic repulsion was the primary stabilization mechanism. However, solvation forces may also play a significant role, and we conducted a homocoagulation analysis of the CNM/ChNC inks with different concentrations of NaCl (Figure S8) to investigate this. Coagulation was observed only at NaCl concentrations above 0.1 mol/L, with partial separation at 0.01 mol/L, suggesting the importance of the interaction between the hybrid and the water. This behavior suggests that the hydrophilic planes of the ChNCs are orientated toward the water, promoting solvation forces that contribute significantly to the colloidal stability due to the hydration force.^39^

**Figure 2:**
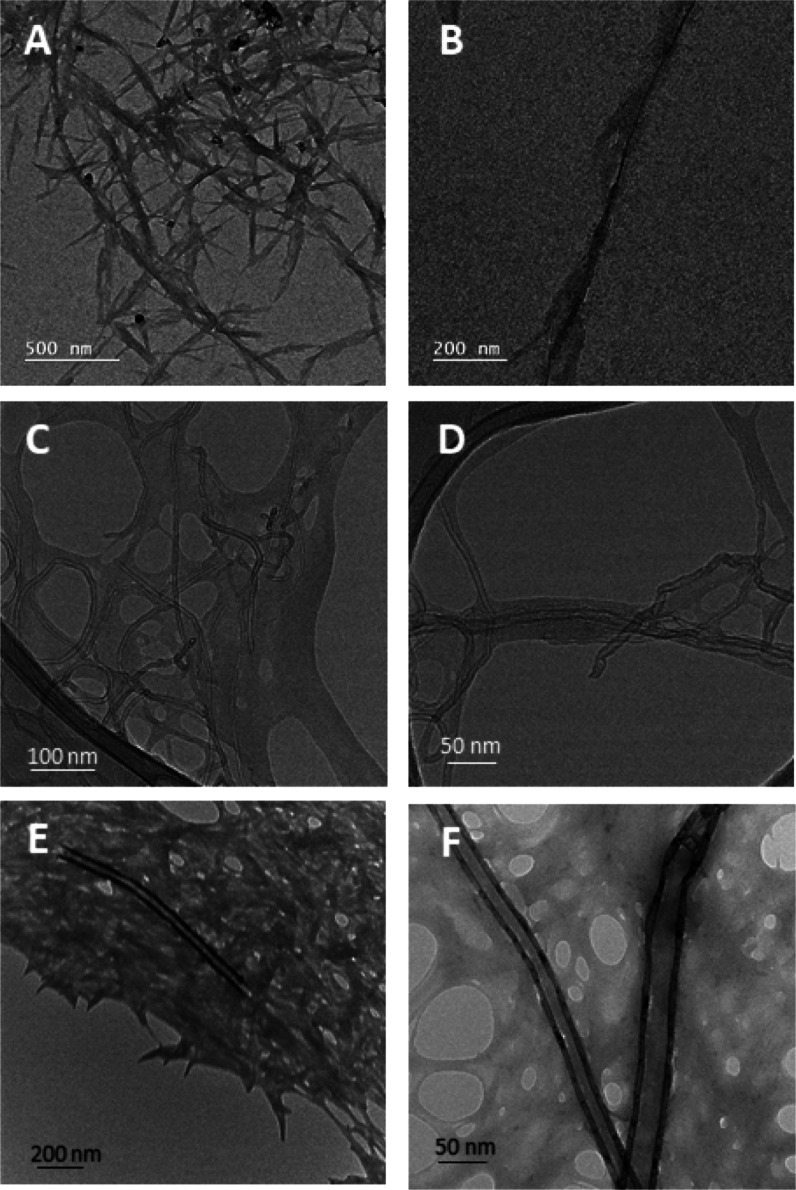
TEM images of the optimal CNM/ChNC inks at different magnifications: SWCNT/ChNC (A,B), MWCNT/ChNC (C,D), and CNF/ChNC (E,F).

The subsequent analysis of the solid material obtained after freeze-drying the optimal inks confirms the effective integration of ChNCs within the hybrid materials and offers insights into the structural features resulting from the interaction between ChNCs and CNMs. The XRD profiles (Figure 3A) of the hybrid materials before any thermal treatment closely resemble that of bare ChNCs (Figure S3A), with slight but significant differences. Interestingly, a broad peak at approximately 26° emerged, indicating an overlap of the contributions from both the (013) plane of chitin and the (002) graphitic plane of the CNMs. Additional changes, such as the reduced intensity of the (021) plane peak and an increased intensity of the (020) plane peak, the latter being more pronounced in the CNFs hybrid, also suggest interactions between ChNCs and CNMs. Notably, the absence of the 6° diffraction peak, corresponding to the SWCNTs bundles packing (Figure S1A), in the SWCNT hybrid (Figure 3A) indicates the disaggregation and the individualization of the SWCNT.^40^ After thermal treatment, the XRD profiles of all three hybrids are very similar, with the peaks corresponding to chitin disappearing, leaving only a broad peak that can be ascribed to a graphitic carbon plane around 26°. Raman spectra of all CNM/ChNC hybrids (Figure 3B) show the characteristic G-band around 1580 cm^–1^, related to the graphitic nature and structural quality of the CNMs, and a strong D-band at 1340 cm^–1^ in both CNF/ChNC and MWCNT/ChNC hybrids associated with the presence of structural defects and disorder in the CNM lattice. Also, the radial breathing modes characteristic of SWCNTs are visible in the low-frequency range of the Raman spectrum before and after the thermal treatment (Figure 3B). No discernible signals from ChNCs appear in the Raman spectra of all samples due to the low Raman resonance, inherent to ChNCs. Interestingly, TGA analysis of the hybrids without thermal treatment (Figure 3C) shows onset degradation temperatures and thermal profiles similar to those of ChNCs (Figure S3C). Furthermore, the mass residue analysis at 800 °C (Figure 3C) highlights the dominance of ChNCs as the primary component of the inks, with the thermal degradation of each CNM being negligible (Figure S1C) compared to that of ChNCs (Figure S3C). Differences in residue values may hint at the ability of ChNCs to stabilize each type of CNM. In particular, the MWCNT/ChNC hybrid exhibits the highest residue (33.9%), followed by the SWCNT/ChNC hybrid (24.2%), indicating a more significant presence of these two CNMs in the inks compared to the CNF/ChNC hybrid, which shows a residue of 18.9%, similar to that of ChNCs alone (16%, Figure S3C). As expected, the TGA analysis of the thermally treated hybrids (Figure 3C) only shows a small mass loss starting at 600 °C due to the previous pyrolysis of most of the ChNCs during the thermal treatment.

**Figure 3:**
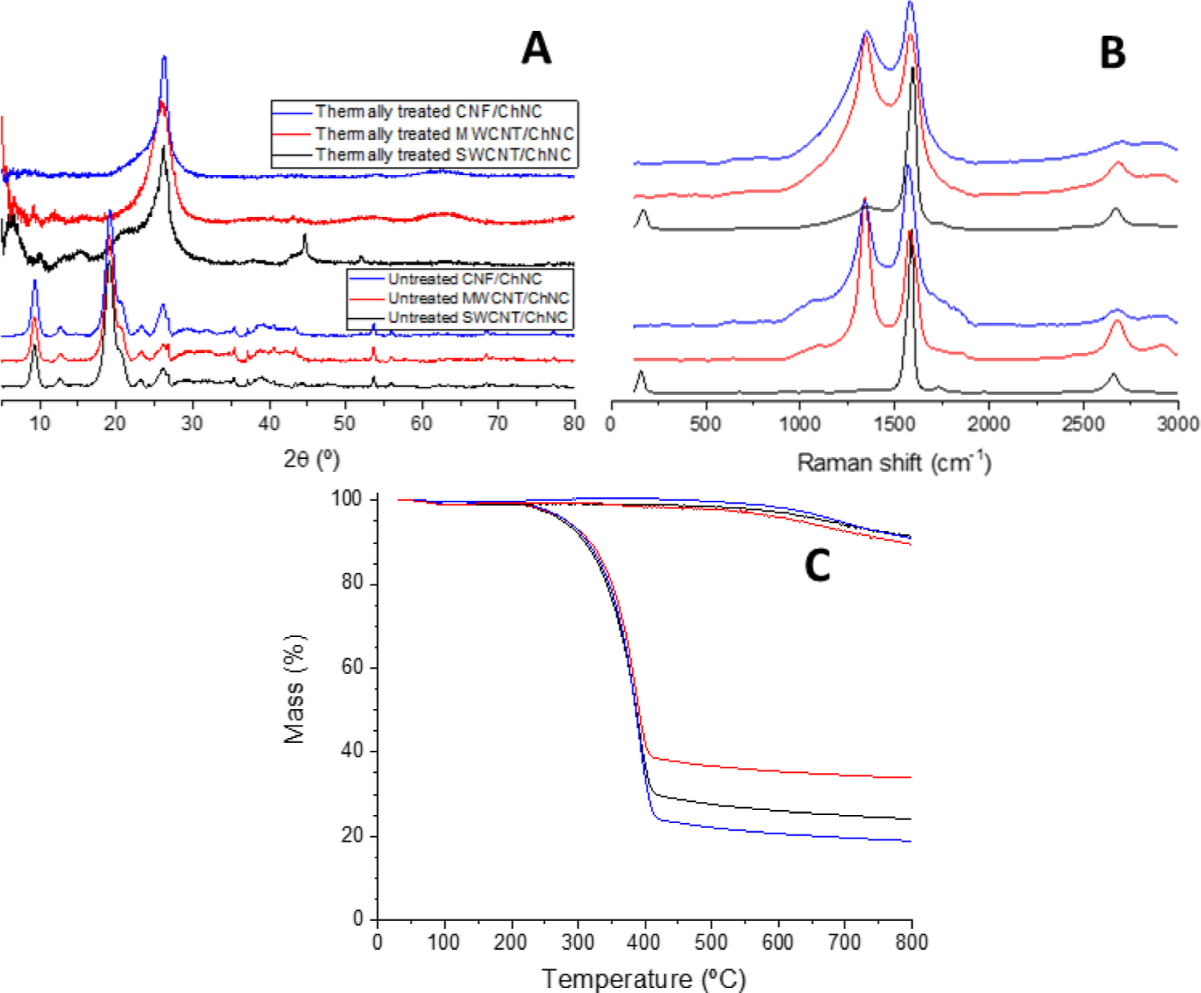
Characterization of CNM/ChNC hybrids prepared from freeze-dried optimal inks by (A) XRD, (B) Raman spectroscopy, and (C) TGA in a N_2_ atmosphere (upper 3 curves correspond to thermally treated samples at 450 °C for 30 min under a N_2_ atmosphere).

The optimized CNM/ChNC inks were used for the development of conductive films by spray coating onto glass substrates. At this point, it is important to recall that the selected formulations were designed to maximize the CNM content, avoiding unnecessary ChNCs surplus. The proposed processing offers significant implications not only for the potential of scalable film fabrication but also for the excellent and precise control of the film thickness and, consequently, for sheet resistance (*R*_S_). Thus, the spray coating parameters were adjusted to reach the lowest possible *R*_S_ values, in line with practical electrical conductivity requirements (Figure 4). However, CNF/ChNCs inks, even if colloidally stable and useful for any desired application in liquid form, produced films with lower adhesion to the substrate and most importantly, significantly higher *R*_S_ (Figure S9) compared to their nanotube analogues. For this reason, the results presented hereafter will focus only on SWCNT and MWCNT formulations.

**Figure 4:**
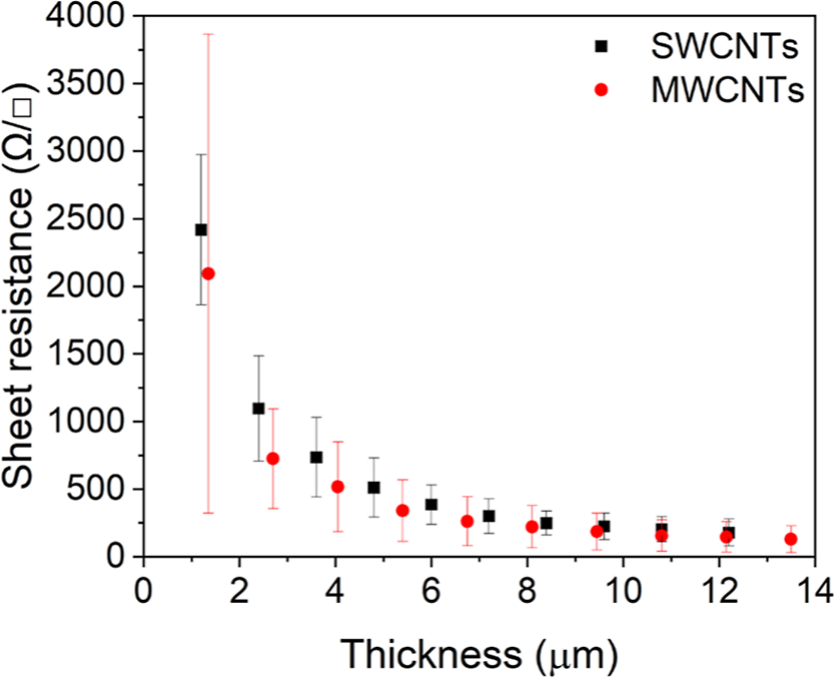
*R*_S_ of samples with increased thickness of SWCNT/ChNC (black squares) and MWCNT/ChNC (red squares) conductive films.

The casting process results in MWCNT-based films with a thickness of 13.5 μm and *R*_S_ of 178 Ω/□ and SWCNT-based films with a thickness of 12.2 μm and *R*_S_ of 129 Ω/□. These *R*_S_ values reassert the successful preparation of conductive CNM-based films stabilized by ChNCs, which are particularly promising for the development of functional carbon-based electrodes, typically characterized by *R*_S_ values within the range of 10 and 1000 Ω/□.^6,35,41^

To improve the electrical characteristics of the films, a thermal treatment under a N_2_ atmosphere was applied. Various characterization techniques were employed to analyze the effects on the properties of both SWCNT/ChNC and MWCNT/ChNC films before and after the thermal treatment. SEM images (Figure S10A,C) suggest that the nanotubes were embedded in ChNCs before the thermal treatment, which increased the electrical conductivity while reducing the film thickness in both cases (Table 1), ending up with more nanotubes exposed (Figure S10B,D). Accordingly, this reduction in film thickness results in enhanced bulk conductivity (Table 1). The *R*_S_ values, ranging from 10 to 1000 Ω/□, are typical for carbon-based electrodes.^6,35,41^ XPS results of the nontreated CNM/ChNC (Figures S11 and S12 and Table S5) also show the high content in chitin and low content of sp^2^ carbon coming from the CNMs (around 5% for the SWCNTs and 12% for the MWCNTs). The XPS measurements after the thermal treatment show the disappearance of all features coming from chitin, the consequent increase of the overall sp^2^ character of the material, and the emergence of new nitrogen and carbon components (namely, pyrrolic and pyridinic nitrogen, some N=O groups, and π–π* shakeup contributions to C 1s). Contact angle measurements of the films with water (Figure S13 and Table S6) reflect a decrease in their hydrophilicity after the thermal treatment. These observations, along with the XRD, Raman spectroscopy, and TGA of the lyophilized inks (Figure 3), confirm the pyrolysis of ChNCs, resulting in films composed of partially naked CNTs with enhanced electrical properties. These encouraging results underscore the feasibility of producing conductive films made of different unfunctionalized carbon nanotubes, facilitated by ChNCs as a processing adjuvant and further improved by pyrolysis.

**Table 1:** Thickness, *R*_s_, and Electrical Conductivity of the Films before and after Thermal Treatment in a N_2_ Atmosphere^a^

films	thickness (μm)	*R*_S_ (Ω/□)	σ (S·cm^–1^)
nontreated SWCNT/ChNC	12.2	129	6.3
treated SWCNT/ChNC	11.6	90	9.5
nontreated MWCNT/ChNC	13.5	178	4.1
treated MWCNT/ChNC	12.3	120	6.7

a Conductivity values are calculated according to eq S2.

After the surface morphology and surface electric properties of SWCNT/ChNC and MWCNT/ChNC conductive films are assessed, the next key step involves the evaluation of their electrochemical behavior, particularly in terms of electronic transfer processes at the electrode/electrolyte interface. This proof of concept is necessary not only for understanding the impact of ChNCs within the system but also for obtaining further insights into how their pyrolysis affects the electrochemical response of the films.

Cyclic voltammetry measurements of the films, both as-prepared and thermally treated (Figure 5), were carried out to evaluate their electrochemical performance in the presence of three different redox probes: ferricyanide, hydroquinone, and ascorbic acid. It should be highlighted that these electrochemical reactions are controlled by diffusion in analogous systems.^42^ The outcomes reveal notable variations in the electrochemical behavior depending on the specific redox probe utilized, emphasizing the complex charge transfer dynamics taking place. The ferricyanide probe follows an outer sphere charge transfer mechanism, in which the redox process occurs between the probe and the electrode with no specific chemical interactions.^35^ As such, the thermal treatment induced an efficiency increase of the electrochemical Faradaic charge transfer, as evidenced by a decrease in the peak-to-peak distance compared to the nontreated samples (Figure 5A,B, and Table 2). Conversely, the cyclic voltammograms using hydroquinone (Figure 5C,D) and ascorbic acid (Figure 5E,F) as redox probes show different electrochemical behavior. In particular, they follow an inner sphere charge transfer mechanism, facilitated by specific interactions with aromatic rings and functional groups on the carbon nanotubes surface.^42,43^ The nontreated electrodes exhibit a strong inhibition when reacting with the electrochemical probes, as no peaks were detected. Notably, the enhanced charge transfer observed after the thermal treatment allows the detection of oxidation and reduction processes for the hydroquinone probe as well as the facile detection of the oxidized form of ascorbic acid, which is a typical example of irreversible electrochemical processes. More specifically, the observed peaks for both hydroquinone and ascorbic acid using the N_2_-treated electrodes appear slightly titled likely due to the remaining pyrolyzed ChNC residue covering portions of the CNT surface. As previously stated, the redox reaction occurs through an inner sphere mechanism that implies direct physical contact between the redox probe and the carbon nanotube surface. The presence of the ChNC pyrolyzed residue can hinder the accessibility of the redox probes to interact with the active sites (such as aromatic rings and functional groups) on the CNTs, thus affecting the voltammogram profiles.

**Figure 5:**
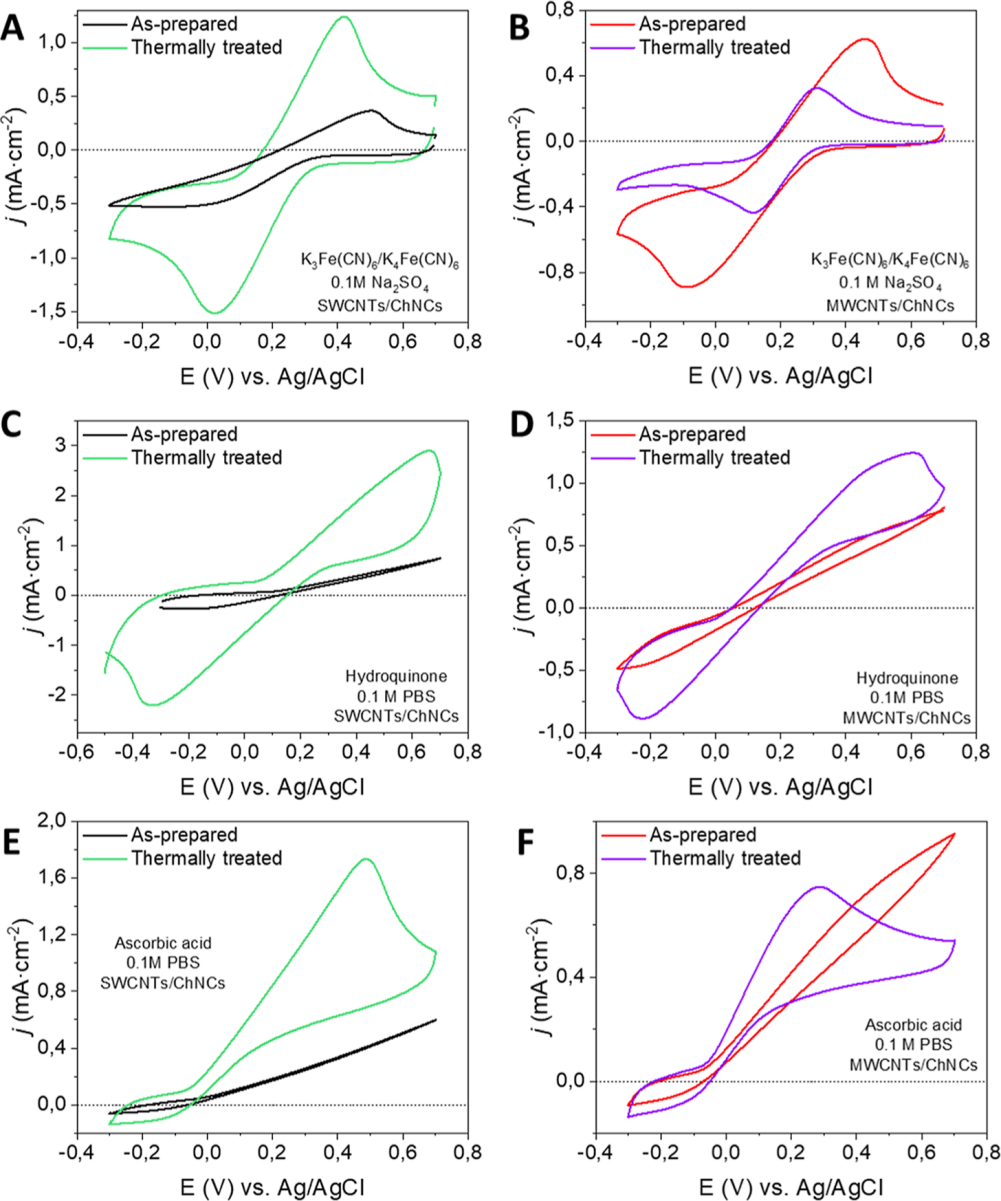
Cyclic voltammograms in the presence of three different redox probes: (A,B) K_3_Fe­(CN)_6_/K_4_Fe­(CN)_6_, (C,D) hydroquinone, and (E,F) ascorbic acid. (A), (C), and (E) correspond to SWCNT/ChNC-based electrodes and (B), (D), and (F) correspond to MWCNT/ChNC-based electrodes. Note that all redox probes were set at 10^–2^ M, and measurements were carried out at a scan rate of 10 mV·s^–1^.

**Table 2:** Voltammetric Peak Separation (Δ*E*_p_) at 10 mV·s^–1^ and the Standard Electrochemical Rate Constant (*k* ^0^) for the Cyclic Voltammograms Involving the Ferro/Ferricyanide, Hydroquinone, and Ascorbic Acid Redox Couples

redox couple	electrode	Δ*E*_p_ (V)	*k*^0^ (cm·s^–1^)
**ferro/ferricyanide**	**nontreated** **SWCNT/ChNC**	0.62	1.8 × 10^–6^
	**treated** **SWCNT/ChNC**	0.4	4.1 × 10^–5^
	**nontreated** **MWCNT/ChNC**	0.55	1.5 × 10^–5^
	**treated** **MWCNT/ChNC**	0.19	3.6 × 10^–4^
**hydroquinone**	**treated** **SWCNT/ChNC**	0.99	1.8 × 10^–12^
	**treated** **MWCNT/ChNC**	0.82	7.0 × 10^–11^
**ascorbic acid**	**treated** **SWCNT/ChNC**		5.7 × 10^–4^
	**treated** **MWCNT/ChNC**		5.1 × 10^–4^

To further investigate the electrochemical properties of the different electrodes, we determined the electron transfer rate constant using the peak separation in the cyclo-voltammograms, according to an expanded numerical solution of Nicholson’s method.^44,45^ The standard electrochemical rate constant (*k* ^0^) was calculated from the cyclic voltammetry measurements (eq S3). Notably, *k* ^0^ values decreased 1 order of magnitude in both SWCNT/ChNC (1.8 × 10^–6^ vs 4.1 × 10^–5^ cm·s^–1^) and MWCNT/ChNC (1.5 × 10^–5^ vs 3.6 × 10^–4^ cm·s^–1^) pyrolyzed electrodes using the ferro/ferricyanide couple compared to their nontreated counterparts (Table 2). The large separation of the voltammogram peaks in the nontreated electrodes (0.62 V in SWCNT/ChNC and 0.55 V in MWCNT/ChNC) evidence the charge transfer blocking at the electrode/electrolyte interface. The inhibition of the outer sphere charge transfer mechanism demonstrates that the presence of ChNCs forms a barrier between the carbon nanotubes and the electrolyte, which matches the SEM and XPS observations (Figures S10,S11 and S12) with the obtained electrochemical results (Figure 5A). This effect is particularly more intense in the case of hydroquinone, in which the reaction is further impeded as it requires the adsorption onto the active sites of carbon nanotubes. In this sense, the calculation of the *k* ^0^ is only performed at the pyrolyzed electrodes (Table 2) as peaks become visible and well-defined (1.8 × 10^–12^ cm·s^–1^ for SWCNT/ChNC and 7.0 × 10^–11^ cm·s^–1^ for MWCNT/ChNC electrodes). The case of irreversible electrochemical reactions is notably different as it requires the use of other equation and parameters for its estimation (eq S4). In this sense, the Nicholson–Shein methodology was followed for the reduction of ascorbic acid, including a modification for determining *k* ^0^ from voltammograms.^46,47^ Again, the calculation of *k* ^0^ for the electrochemical reduction of ascorbic acid is limited to the N_2_-treated electrodes due to the clear appearance of the peak. The obtained value for the MWCNTs/ChNCs electrode is 5.7 × 10^–4^ cm·s^–1^, while the SWCNTs/ChNCs displays a *k* ^0^ of 5.1 × 10^–4^ cm·s^–1^. In general, the calculated *k* ^0^ values fall within the range of those observed for other electrode materials,^35,41^ highlighting the considerable advantage of integrating ChNCs into CNMs processing. Such an improvement in the charge transfer properties of the pyrolyzed electrodes could be explained according to two main findings: on the one hand, carbon nanotubes are more exposed to the electrolyte after pyrolysis, and on the other hand, a new CNT/ChNC hybrid is formed upon thermal treatment, which agrees with the XPS observations.^43^

## Conclusions

Our research reveals exciting prospects for employing ChNCs, synthesized via acid hydrolysis, for stabilizing three different types of 1D CNMs in water. We successfully stabilized SWCNTs, MWCNTs, and CNFs at concentrations up to 0.74 0.58, and 0.44 g/L, respectively. We propose their use as inks in the fabrication of carbon-based films on glass substrates via spray coating, a controllable and scalable processing technique. Surprisingly, initial films containing insulating ChNCs exhibited a low sheet resistance. To investigate how ChNCs influence conductive films, we thermally treated the films under a nitrogen atmosphere. This post-treatment significantly enhanced the electrical conductivity of the films, reaching 9.5 S·cm^–1^ for SWCNT films and 6.7 S·cm^–1^ for MWCNT films. Electrochemical characterization of such films revealed that ChNCs removal during pyrolysis has important implications for charge transfer processes, improving the ability to transfer electrons at the electrode/electrolyte interface. This was evidenced by the lower peak separation and faster reaction rates of the pyrolyzed electrodes. We suggest that such an improvement is due to increased accessibility of the electrolyte to the carbon nanotubes and to the formation of a new hybrid upon pyrolysis, supported by structural and surficial characterization. Overall, our findings highlight the potential of ChNCs as a green and effective processing adjuvant for 1D CNMs and could be extended to other CNMs and nanomaterials in general. This approach allows for the development of water-based conductive inks, films, and electrodes with promise across a broad spectrum of applications from electronics to energy storage, emphasizing the importance of sustainable development in functional device components.

## Supplementary Material



## Notes

Additional experimental details, including the surface conditioning of CNMs and the characterization of CNMs and ChNCs along with their combinations, further information about the different CNM/ChNC inks behavior and their subsequent films, and calculations regarding the CNM concentration as well as the electrical conductivity and the standard electrochemical constant (PDF)

## References

[1] Istif E., Hernández-Ferrer J., Urriolabeitia E. P., Stergiou A., Tagmatarchis N., Fratta G., Large M. J., Dalton A. B., Benito A. M., Maser W. K. (2018). Conjugated Polymer
Nanoparticle–Graphene Oxide Charge-Transfer Complexes. Adv. Funct. Mater..

[2] Ansón-Casaos A., Hernández-Ferrer J., Vallan L., Xie H., Lira-Cantú M., Benito A. M., Maser W. K. (2021). Functionalized Carbon
Dots on TiO_2_ for Perovskite Photovoltaics and Stable Photoanodes
for Water Splitting. Int. J. Hydrogen Energy.

[3] González-Domínguez J., Grasa L., Frontiñán-Rubio J., Abás E., Domínguez-Alfaro A., Mesonero J. E., Criado A., Ansón-Casaos A. (2022). Intrinsic and Selective Activity
of Functionalized Carbon Nanotube/Nanocellulose Platforms against
Colon Cancer Cells. Colloids Surf., B.

[4] Souza V. H. R., Husmann S., Neiva E. G. C., Lisboa F. S., Lopes L. C., Salvatierra R. V., Zarbin A. J. G. (2016). Flexible, Transparent and Thin Films
of Carbon Nanomaterials as Electrodes for Electrochemical Applications. Electrochim. Acta.

[5] Ansón-Casaos A., Mis-Fernández R., López-Alled C.
M., Almendro-López E., Hernández-Ferrer J., González-Domínguez J. M., Martínez M. T. (2015). Transparent Conducting Films Made of Different Carbon
Nanotubes, Processed Carbon Nanotubes, and Graphene Nanoribbons. Chem. Eng. Sci..

[6] González-Domínguez J. M., Baigorri A., Álvarez-Sánchez M. Á., Colom E., Villacampa B., Ansón-Casaos A., García-Bordejé E., Benito A. M., Maser W. K. (2021). Waterborne
Graphene- and Nanocellulose-Based Inks for Functional Conductive Films
and 3D Structures. Nanomaterials.

[7] Schroeder V., Savagatrup S., He M., Lin S., Swager T. M. (2019). Carbon
Nanotube Chemical Sensors. Chem. Rev..

[8] Shahzad N., Lutfullah, Perveen T., Pugliese D., Haq S., Fatima N., Salman S. M., Tagliaferro A., Shahzad M. I. (2022). Counter Electrode Materials Based
on Carbon Nanotubes for Dye-Sensitized Solar Cells. Renewable Sustainable Energy Rev..

[9] Reza M. S., Afroze S., Kuterbekov K., Kabyshev A., Bekmyrza K. Z., Haque M. N., Islam S. N., Hossain M. A., Hassan M., Roy H., Islam M. S., Pervez M. N., Azad A. K. (2023). Advanced Applications
of Carbonaceous Materials in Sustainable Water Treatment, Energy Storage,
and CO2 Capture: A Comprehensive Review. Sustainability.

[10] Santos F., Lorca S., Gonzalez-Martinez J.
F., Urbina A., Alvarez-Sanchez M. A., González-Domínguez J. M., García-Bordejé E., Ansón-Casaos A., Benito A. M., Maser W. K., Fernández Romero A. J. (2024). Metal-free
Nanostructured-carbon Inks for a Sustainable Fabrication of Zinc/Air
Batteries: From ORR Activity to a Simple Prototype. Appl. Res..

[11] Lu S., Smith B. N., Meikle H., Therien M. J., Franklin A. D. (2023). All-Carbon
Thin-Film Transistors Using Water-Only Printing. Nano Lett..

[12] Calvo V., Paleo A. J., González-Domínguez J. M., Muñoz E., Krause B., Pötschke P., Maser W. K., Benito A. M. (2024). The Aqueous Processing of Carbon
Nanofibers via Cellulose Nanocrystals as a Green Path towards E-Textiles
with n-Type Thermoelectric Behaviour. Carbon.

[13] Dortez S., Sierra T., Álvarez-Sánchez M. Á., González-Domínguez J. M., Benito A. M., Maser W. K., Crevillen A. G., Escarpa A. (2022). Effect of Nanocellulose Polymorphism
on Electrochemical Analytical Performance in Hybrid Nanocomposites
with Non-Oxidized Single-Walled Carbon Nanotubes. Microchim. Acta.

[14] Shamshina J. L., Berton P., Rogers R. D. (2019). Advances
in Functional Chitin Materials:
A Review. ACS Sustain. Chem. Eng..

[15] Jin T., Liu T., Lam E., Moores A. (2021). Chitin and Chitosan on the Nanoscale. Nanoscale Horiz..

[16] Bai L., Liu L., Esquivel M., Tardy B. L., Huan S., Niu X., Liu S., Yang G., Fan Y., Rojas O. J. (2022). Nanochitin: Chemistry,
Structure, Assembly, and Applications. Chem.
Rev..

[17] Salaberria A. M., Labidi J., Fernandes S. C. M. (2015). Different Routes to Turn Chitin into
Stunning Nano-Objects. Eur. Polym. J..

[18] Yang T., Qi H., Liu P., Zhang K. (2020). Selective Isolation Methods for Cellulose
and Chitin Nanocrystals. ChemPlusChem.

[19] Muñoz-Núñez C., Fernández-García M., Muñoz-Bonilla A. (2022). Chitin Nanocrystals:
Environmentally Friendly Materials for the Development of Bioactive
Films. Coatings.

[20] Yan Y., Ge F., Qin Y., Ruan M., Guo Z., He C., Wang Z. (2020). Ultralight and Robust Aerogels Based on Nanochitin
towards Water-Resistant
Thermal Insulators. Carbohydr. Polym..

[21] Calvo V., Fuentes L., Berdejo D., González-Domínguez J. M., Maser W. K., Benito A. M. (2023). Oil-in-Water
Pickering Emulsions
Stabilized with Nanostructured Biopolymers: A Venue for Templating
Bacterial Cellulose. Int. J. Mol. Sci..

[22] Tzoumaki M. V., Moschakis T., Kiosseoglou V., Biliaderis C. G. (2011). Oil-in-Water
Emulsions Stabilized by Chitin Nanocrystal Particles. Food Hydrocolloids.

[23] Salaberria A. M., Labidi J., Fernandes S. C. M. (2014). Chitin
Nanocrystals and Nanofibers
as Nano-Sized Fillers into Thermoplastic Starch-Based Biocomposites
Processed by Melt-Mixing. Chem. Eng. J..

[24] Ifuku S., Saimoto H. (2012). Chitin Nanofibers:
Preparations, Modifications, and
Applications. Nanoscale.

[25] González-Domínguez J. M., Ansón-Casaos A., Grasa L., Abenia L., Salvador A., Colom E., Mesonero J. E., García-Bordejé J. E., Benito A. M., Maser W. K. (2019). Unique Properties and Behavior of
Nonmercerized Type-II Cellulose Nanocrystals as Carbon Nanotube Biocompatible
Dispersants. Biomacromolecules.

[26] Narkevicius A., Steiner L. M., Parker R. M., Ogawa Y., Frka-Petesic B., Vignolini S. (2019). Controlling
the Self-Assembly Behavior of Aqueous Chitin
Nanocrystal Suspensions. Biomacromolecules.

[27] Sèbe G., Ham-Pichavant F., Ibarboure E., Koffi A. L. C., Tingaut P. (2012). Supramolecular
Structure Characterization of Cellulose II Nanowhiskers Produced by
Acid Hydrolysis of Cellulose I Substrates. Biomacromolecules.

[28] He Y., Lin X., Feng Y., Luo B., Liu M. (2022). Carbon Nanotube Ink
Dispersed by Chitin Nanocrystals for Thermoelectric Converter for
Self-Powering Multifunctional Wearable Electronics. Advanced Science.

[29] Carbon solutions Inc . P2-SWNT. 2023. https://www.carbonsolution.com/products/p2-swnt (accessed 10 13, 2023).

[30] Nanocyl . NC7000. 2023. https://www.nanocyl.com/product/nc7000/(accessed 05 12, 2023).

[31] Paleo A. J., Vieira E. M. F., Wan K., Bondarchuk O., Cerqueira M. F., Bilotti E., Melle-Franco M., Rocha A. M. (2020). Vapor Grown Carbon Nanofiber Based Cotton Fabrics with
Negative Thermoelectric Power. Cellulose.

[32] Martínez M. T., Callejas M. A., Benito A. M., Cochet M., Seeger T., Ansón A., Schreiber J., Gordon C., Marhic C., Chauvet O., Fierro J. L. G., Maser W. K. (2003). Sensitivity of Single
Wall Carbon Nanotubes to Oxidative Processing: Structural Modification,
Intercalation and Functionalisation. Carbon.

[33] González-Domínguez J. M., Castell P., Ansón A., Maser W. K., Benito A. M., Martinez M. T. (2009). Block Copolymer Assisted Dispersion of Single Walled
Carbon Nanotubes and Integration into a Trifunctional Epoxy. J. Nanosci. Nanotechnol..

[34] Choi W. K., Park S. G., Takahashi H., Cho T. H. (2003). Purification of
Carbon Nanofibers with Hydrogen Peroxide. Synth.
Met..

[35] Ansón-Casaos A., Sanahuja-Parejo O., Hernández-Ferrer J., Benito A. M., Maser W. K. (2020). Carbon
Nanotube Film Electrodes with Acrylic Additives:
Blocking Electrochemical Charge Transfer Reactions. Nanomaterials.

[36] Smith M., Scudiero L., Espinal J., McEwen J. S., Garcia-Perez M. (2016). Improving
the Deconvolution and Interpretation of XPS Spectra from Chars by
Ab Initio Calculations. Carbon.

[37] Itkis M. E., Perea D. E., Niyogi S., Rickard S. M., Hamon M. A., Hu H., Zhao B., Haddon R. C. (2003). Purity Evaluation of As-Prepared
Single-Walled Carbon Nanotube Soot by Use of Solution-Phase near-IR
Spectroscopy. Nano Lett..

[38] Ansón-Casaos A., Ciria J. C., Sanahuja-Parejo O., Víctor-Román S., González-Domínguez J. M., García-Bordejé E., Benito A. M., Maser W. K. (2020). The Viscosity of Dilute Carbon Nanotube
(1D) and Graphene Oxide (2D) Nanofluids. Phys.
Chem. Chem. Phys..

[39] Liang Y., Hilal N., Langston P., Starov V. (2007). Interaction Forces
between Colloidal Particles in Liquid: Theory and Experiment. Adv. Colloid Interface Sci..

[40] Thess A., Lee R., Nikolaev P., Dai H., Petit P., Robert J., Xu C., Lee Y. H., Kim S. G., Rinzler A. G., Colbert D. T., Scuseria G. E., Tománek D., Fischer J. E., Smalley R. E. (1996). Crystalline
Ropes of Metallic Carbon Nanotubes. Science.

[41] Liao Y., Zhang R., Wang H., Ye S., Zhou Y., Ma T., Zhu J., Pfefferle L. D., Qian J. (2019). Highly Conductive Carbon-Based
Aqueous Inks toward Electroluminescent Devices, Printed Capacitive
Sensors and Flexible Wearable Electronics. RSC
Adv..

[42] Sieben J. M., Ansón-Casaos A., Montilla F., Martínez M. T., Morallón E. (2014). Electrochemical
Behaviour of Different Redox Probes
on Single Wall Carbon Nanotube Buckypaper-Modified Electrodes. Electrochim. Acta.

[43] Martín A., Hernández-Ferrer J., Vázquez L., Martínez M. T., Escarpa A. (2014). Controlled Chemistry
of Tailored
Graphene Nanoribbons for Electrochemistry: A Rational Approach to
Optimizing Molecule Detection. RSC Adv..

[44] Nicholson R. S. (1965). Theory
and Application of Cyclic Voltammetry for Measurement of Electrode
Reaction Kinetics. Anal. Chem..

[45] Mahé E., Devilliers D., Comninellis C. (2005). Electrochemical Reactivity at Graphitic
Micro-Domains on Polycrystalline Boron Doped Diamond Thin-Films Electrodes. Electrochim. Acta.

[46] Nicholson R. S., Shain I. (1964). Theory of Stationary
Electrode Polarography. Single Scan and Cyclic
Methods Applied to Reversible, Irreversible, and Kinetic Systems. Anal. Chem..

[47] Velasco J. G. (1997). Determination
of Standard Rate Constants for Electrochemical Irreversible Processes
from Linear Sweep Voltammograms. Electroanal.

