# Supplementary material for: Carbon nanomaterials-Based Inks and Electrodes Using Chitin Nanocrystals
Source: ACS Sustain Chem Eng. 2024 Oct 14;12(43):15980–90. doi: 10.1021/acssuschemeng.4c05253 (PMC12308767; doi:10.1021/acssuschemeng.4c05253)
Supplement: Supplementary file 1 [file sc4c05253_si_001.pdf]

Supporting information

Carbon nanomaterials-based inks and electrodes using chitin nanocrystals

Víctor Calvo,[†] Carlos Martínez-Barón,[†] Benjamín Vázquez-Conejo, [†] Antonio Dominguez-Alfaro,[‡] Antonio J. Paleo,[§] Belén Villacampa,[¶] Alejandro Ansón-Casaos, Wolfgang K. Maser, [†] Ana M. Benito[††] José M. González-Domínguez, [†*]*

[†]Instituto de Carboquímica (ICB-CSIC), C/ Miguel Luesma Castán 4, 50018 Zaragoza, Spain

[‡]Faculty of Chemistry, University of Basque Country, Paseo Manuel Lardizabal 3, 20018 Donostia-San Sebastián, Spain

[§]2C2T-Centre for Textile Science and Technology, University of Minho, Campus de Azurém, 4800-058 Guimarães, Portugal

[¶]Departamento de Física de la Materia Condensada, INMA-CSIC, Universidad de Zaragoza, 50009, Zaragoza, Spain

Supporting information contents:

Number of pages: 24

Number of figures: S1-S13

Number of tables: S1-S5

Number of equations: S1-S4

Table of contents

1. Surface conditioning of CNMs and characterization..S3

2. Calculation of the CNMs concentration post centrifugation S6

3. Dynamic light scattering results of fresh and redispersed ChNCs........................S7

4. Morphological characterization of ChNCs..S8

5. Sheet resistance results of the SWCNT/ChNC films...S10

6. Characterization of the CNM/ChNC dispersions...S11

7. Purity index of SWCNT/ChNC dispersions S13

8. Homocoagulation analysis of SWCNT/ChNC and MWCNT/ChNC dispersions..S15

9. Sheet resistance results of the CNM/ChNC films...S16

10. SEM images of the CNM/ChNC films S17

11. Calculation of the CNM/ChNC films electrical conductivity.............................S18

12. XPS measurements of the CNM/ChNC films..S19

13. Contact angle measurements of the CNM/ChNC films S22

14. Calculation of the standard electrochemical rate constant..............................S23

15. References S25

1. Surface conditioning of CNMs and characterization

Owing to its intrinsic properties, the hydrophobicity of CNMs is the main drawback towards the development of stable aqueous inks, chemical low functionalization is a viable way to improve the hydrophilicity of the CNM surface.[1] However, the majority of purification and covalent functionalization processes are detrimental to the sp^2 conjugation, thus altering and worsening the electronic features of CNMs,[2] highly sought in this work. Bearing this in mind, specific surface conditioning treatments were selected for each CNM to improve its hydrophilicity with minimal modification of their electronic properties. The mass yield of the surface conditioning processes was 97% and 98% for the MWCNTs and the CNFs, respectively. The employed CNMs were characterized before and after purification by four different techniques: X- Ray diffraction (XRD), Raman spectroscopy, thermogravimetric analysis (TGA) and X-Ray photoelectron spectroscopy (XPS). The final residue in the TGA (Figure S1) was 95.8% for the P2-SWCNTs, 96.4% for the purified MWCNTs and 99.1% for the purified CNFs, what indicates their high thermal stability and low-functionalization.

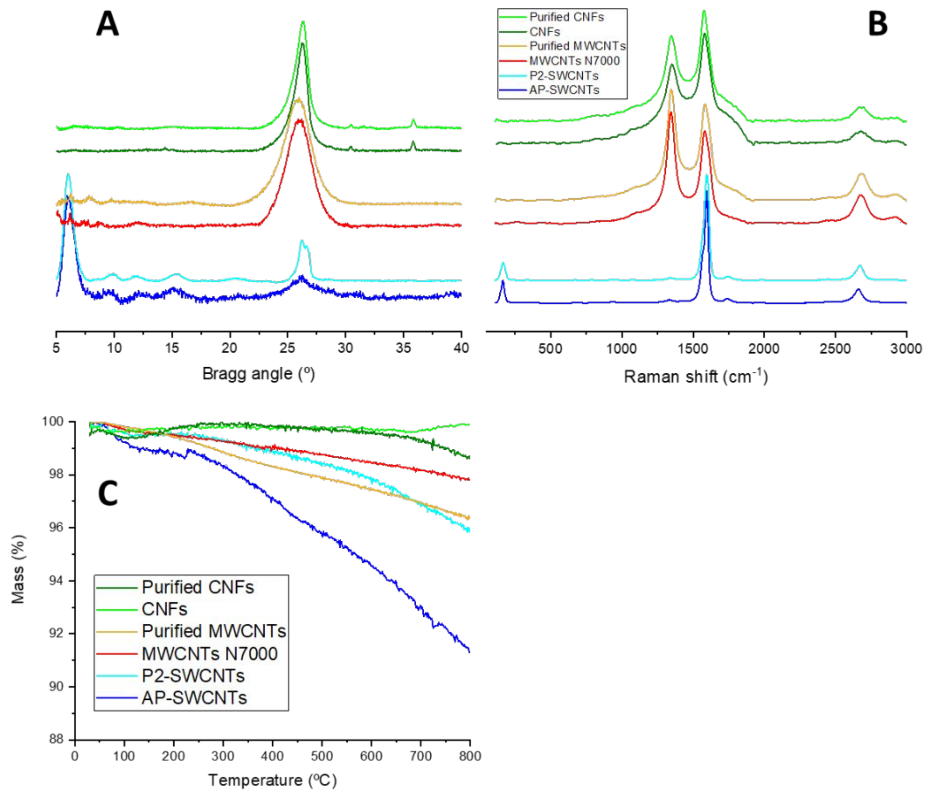

Figure S1. CNMs characterization before and after purification: (A) XRD, (B) Raman spectroscopy and (C) TGA in nitrogen.

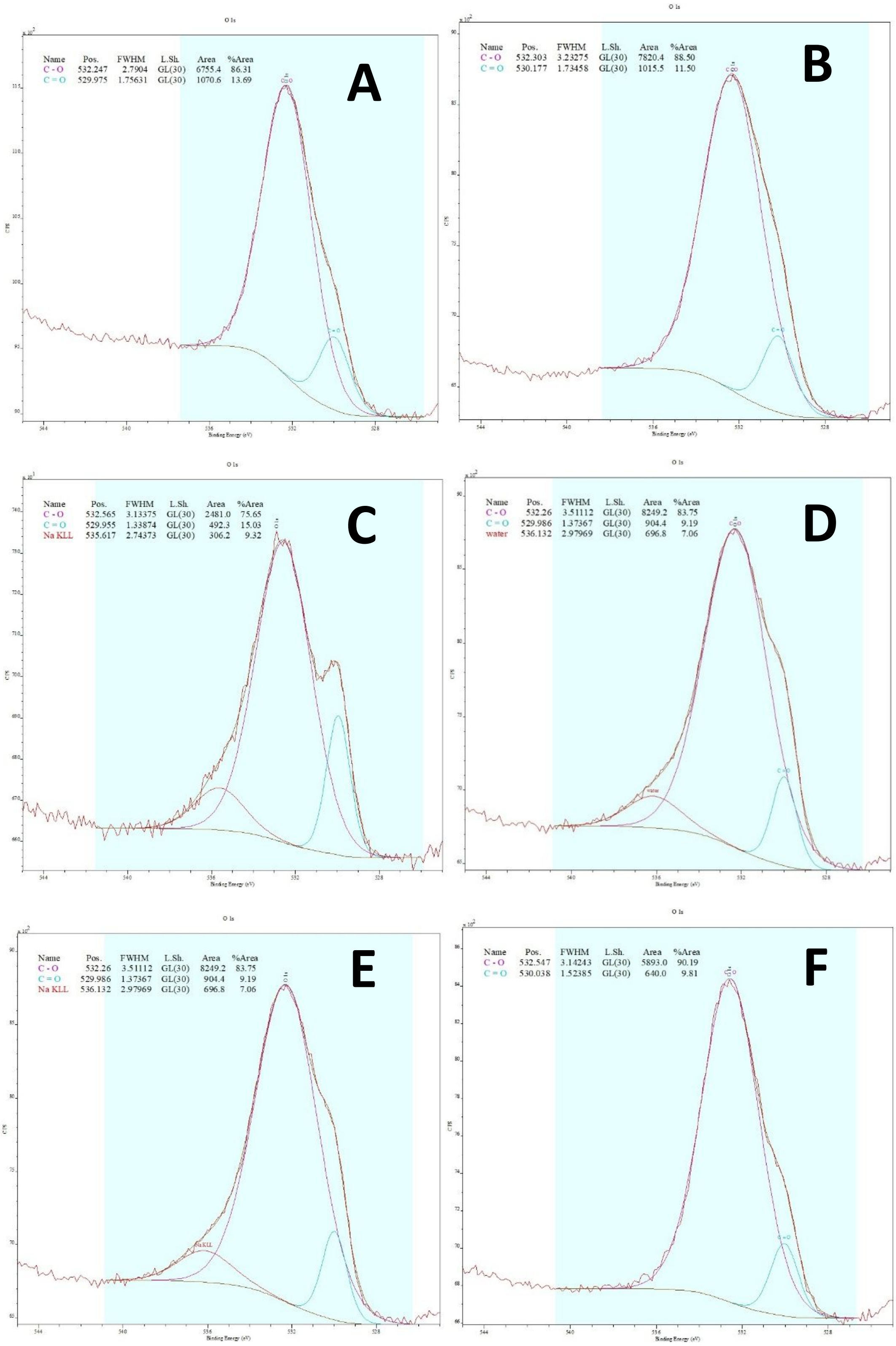

Figure S2. XPS results of the different CNMs before and after purification: (A) AP-SWCNTs, (B) P2-SWCNTs, (C) Nanocyl NC 7000T MWCNTs, (D) purified MWCNTs, (E) Pyrograf®-III PR 24 LHTXT CNFs and (F) purified CNFs.

Table S1. XPS results of CNMs before and after purification

**Sample**	**EA [at.%]**		**O 1s component [area %]**		
	O 1s	C 1s	C-O (532 ± 0.3)[a]	C = O (530.0 ± 0.2)	Na KLL (535.9 ± 0.3)
AP-SWCNTs[b]	3.4	95.7	86.3	13.7	-
SWCNTs-P2	4.4	95.6	88.5	11.5	-
MWCNTs	1.7	98.3	75.6	15.0	9.3
MWCNTs-p	4.0	96.0	83.7	9.2	7.1
CNFs	1.3	98.7	92.1	3.4	4.6
CNFs-p	2.9	97.1	90.2	9.8	-

[a] wide band probably including several band components

[b] additionally detected 0.1% Ni and 0.8% Y

2. Calculation of the CNMs concentration post centrifugation

Equation S1 was employed to obtain the CNMs concentration after centrifugation. Basically, it is based on a comparison between the absorbance values before and after centrifugation, including their corresponding dilution factors. It is important to note that the initial CNMs in all dispersions is 1 g/L.

$$[CNM]_{post\ centrifugation} = \frac{Abs_{post}}{Abs_{pre}} \times \frac{[CNM]_{pre}}{Dilution\ factor_{pre}} \times Dilution\ factor_{post} \qquad (S1)$$

Equation S1. **Calculation of the CNMs concentration post centrifugation.**

$[CNM]_{post\ centrifugation}$: Concentration carbon nanomaterial after centrifugation

Abs_{post}: Absorbance after centrifugation

Abs_{pre}: Absorbance before centrifugation

$[CNM]_{pre\ centrifugation}$: Concentration carbon nanomaterial before centrifugation

$Dilution\ factor_{post}$: Dilution factor of the sample after centrifugation

$Dilution\ factor_{pre}$: Dilution factor of the sample before centrifugation.

3. Dynamic light scattering results of fresh and redispersed ChNCs

Freshly prepared ChNCs aqueous colloids show remarkable stability, underscored by a high ζ-potential value (45 ± 4 mV) and small mean hydrodynamic radius (115 ± 11 nm, with a polydispersity of 0.39 ± 0.08) as determined by DLS analysis (Table S2). Comparing with previous work by Narkevicius et al.,[3] we have obtained a narrower polydispersity, while maintaining similar average size and ζ-potential. The capability of freeze-dried ChNCs to be redispersed in water was tested by preparing a 2 g/L ChNCs dispersion in ultrapure water. An ultrasound bath and sonic tip (in the same conditions above stated) were used to redisperse the ChNCs. The resulting dispersions were measured by DLS and ζ-potential and were compared to the ones of the fresh ChNCs colloids. It was found that results do not significantly differ (Table S2). To the best of our knowledge, such remarkable property is unique to ChNCs, as cellulose nanocrystals cannot be easily redispersed in water and are very sensitive to the sample moisture.[4] As such, this is another reason that supports our aim to use ChNCs as CNM green dispersant, as it maintains its colloidal parameters even after being freeze-dried and stored.

Table S2. DLS results (hydrodynamic radii (R_H)) and ζ-potential of fresh ChNCs dispersions and re-dispersed by ultrasound bath and ultrasound bath + ultrasonic tip

Batch	Freshly prepared		Re-dispersed by ultrasound bath		Re-dispersed by ultrasound bath and ultrasonic tip	
	R_H (nm)	ζ-potential (mV)	R_H (nm)	ζ-potential (mV)	R_H (nm)	ζ-potential (mV)
ChNCs 1	94	44	109	42	54	41
ChNCs 2	116	46	161	44	63	39
ChNCs 3	123	49	394	36	130	34
ChNCs 4	120	49	115	32	71	40
ChNCs 5	125	42	2230	36	125	33
ChNCs 6	110	39	444	37	77	38

4. Morphological characterization of ChNCs

The mean mass yield obtained was (70.1 ± 3.3) %, which is a consistent value according to previous works about ChNCs synthesis by acid hydrolysis with HCl.[3] XRD of the lyophilized ChNCs solid exhibits a main peak at around 19° attributed to the (110) plane, along with a shoulder at 21° ascribed to the (120) plane (Figure S3A), as typically observed in chitin materials.[5] Moreover, a characteristic peak, located below 10°, corresponds to the (020) crystallographic plane. FTIR spectroscopy analysis (Figure S3B) shows the intrinsic vibrational modes of the chitin structure, including the broad O-H bond resonance at 3400 cm^{-1}, the C=O double bond at 1650 cm^{-1}, and the C-N bond at 1560 cm^{-1}, along with multiple N-H bonds within the region between 3100 and 3300 cm^{-1}.[6,7] Thermal degradation profile in a N_2 atmosphere (Figure S3C) shows moisture removal between 25°C and 120°C, with the maximum weight loss centered at 375°C, leaving a residual mass of 16% at 800°C. TEM Images (Figure S3D) corroborate the synthesis of chitin nanocrystals, revealing their typical rod-like morphology, with a high aspect ratio, an average length of approximatively 300-500 nm, and a diameter ranging from 20 to 40 nm.

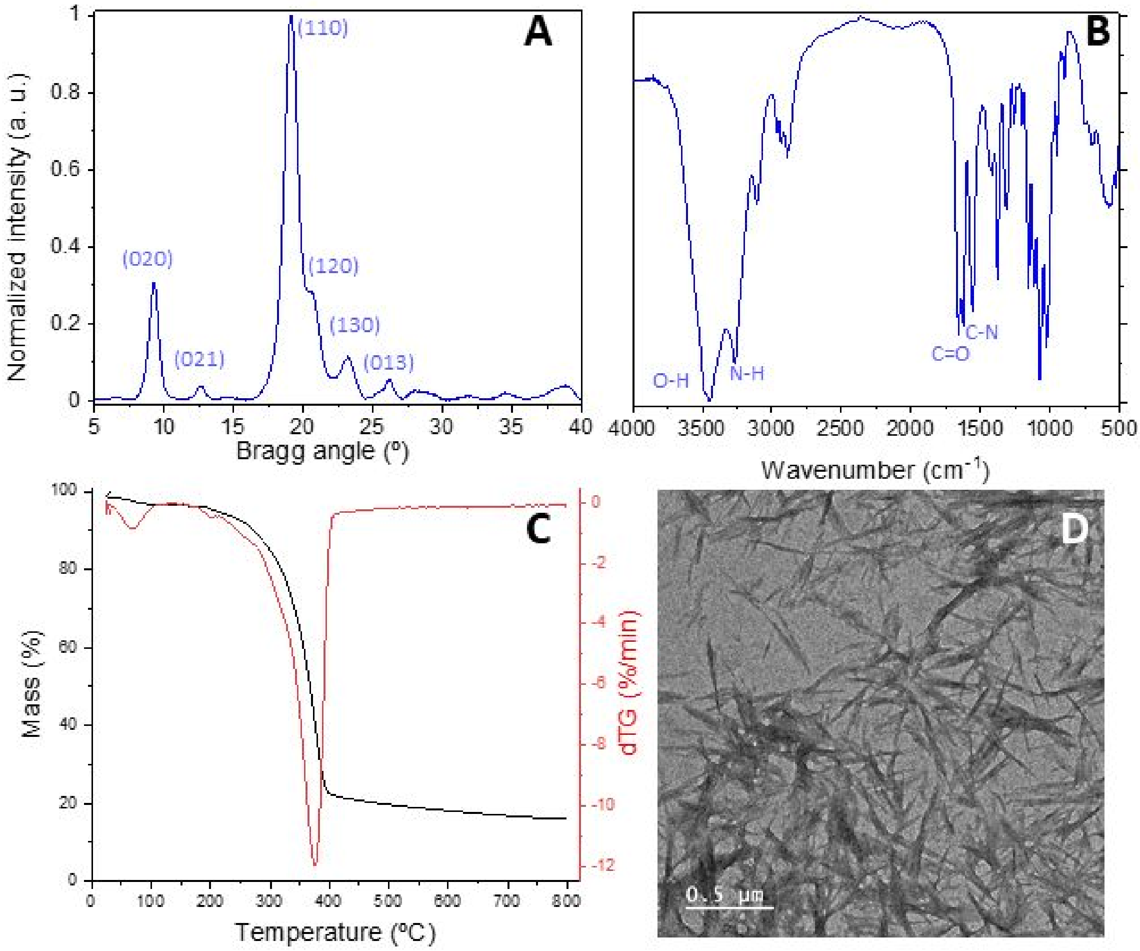

Figure S3. ChNCs characterization: (A) X Ray diffractogram, (B) FTIR spectrum, (C) TGA plot, and (D) a representative TEM image (scale bar = 0.5 µm).

5. Sheet resistance results of the SWCNT/ChNC with different ChNCs concentrations

As commented in the main article, ChNCs concentration is a key parameter ruling the electrical properties of conductive films. Figure S3 displays the different sheet resistance (R_S) values of conductive films made from different ChNCs concentration. Due to the similar results obtained for the optimization of the SWCNT/ChNC inks shown in Figure 3, we decided to further study different proportions of such hybrid. The results (Figure S4) show clearly that the 3 g/L is the optimal formulation for the development of conductive films with this CNM, while exhibiting good colloidal parameters (Figure 3).

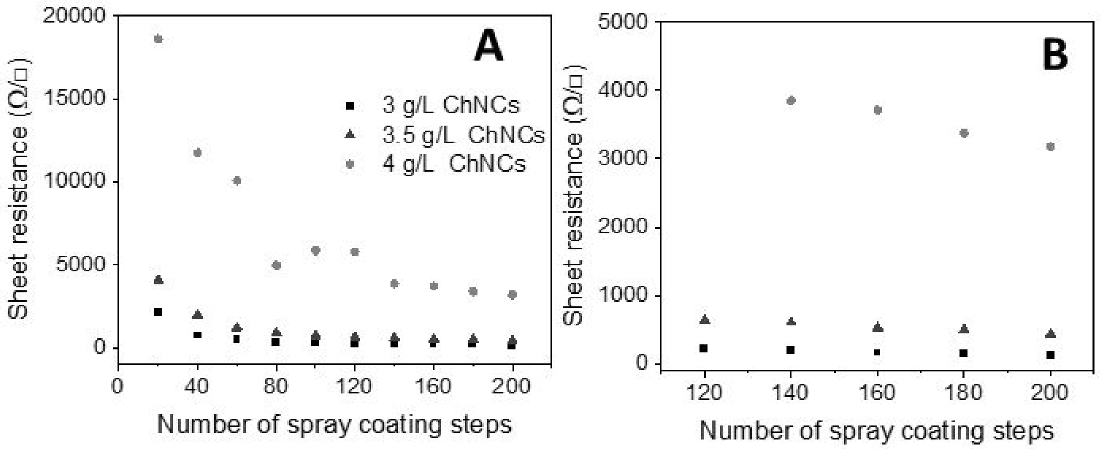

Figure S4. R_S of samples increasing the number of spray coating steps for SWCNT/ChNC inks with different ChNCs concentrations: 3 g/L (black squares), 3.5 g/L (grey triangles) and 4 g/L (grey circles). (A) Complete range and (B) zoom-in from 120 to 200 spray coating steps.

6. Characterization of the CNM/ChNC dispersions

The three optimized CNM/ChNC dispersions have a similar and homogeneous black color, with a high opacity even after centrifugation (Figure S5). Diluted samples before and after centrifugation of these dispersions were measured by UV-vis spectroscopy (Figure S6) to determine the concentration with the equation S1.

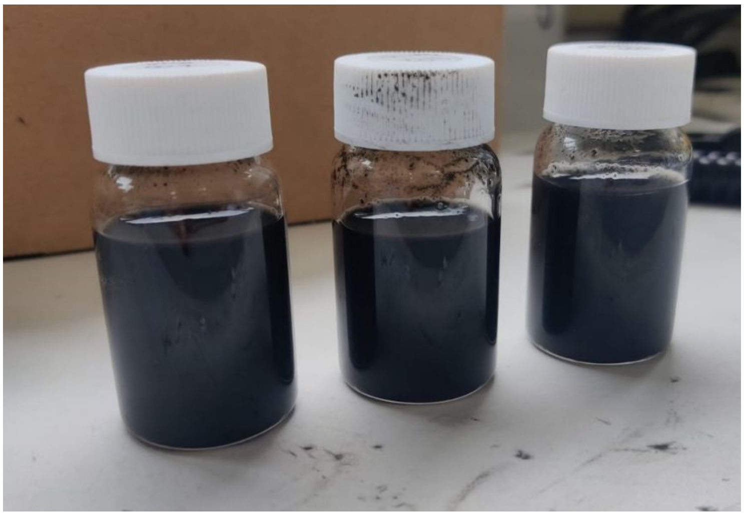

Figure S5. Visual appearance of the optimized dispersions: SWCNT/ChNC (left), MWCNT/ChNC (center) and CNF/ChNC (right).

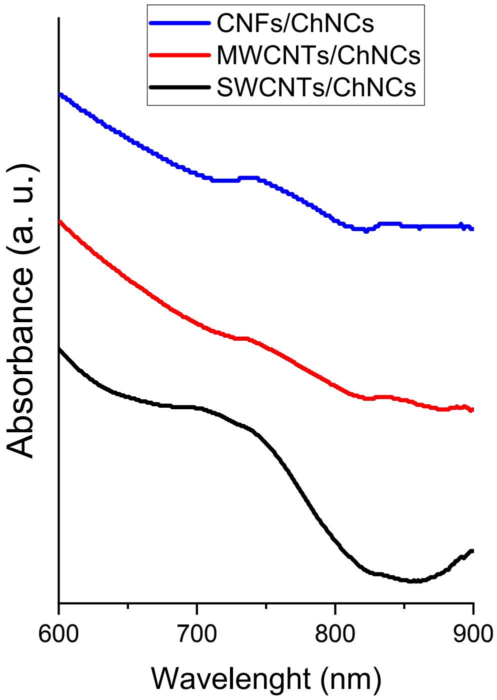

Figure S6. UV-vis absorbance spectra of the optimized CNM/ChNC inks after centrifugation between 600 and 900 nm.

Table S3. Kinematic viscosity values of each CNM/ChNC dispersion at 30 °C. Note that the theoretical kinematic viscosity of water at such temperature is 0.80 mm^2/s.

	H_2O	SWCNT/ChNC	MWCNT/ChNC	CNF/ChNC
**υ (mm^2/s)**	0.79	0.99	0.91	0.86

7. Purity index of SWCNT/ChNC dispersions

NIR spectra of SWCNT/ChNC inks centrifugated at different conditions reveal notable variations. The purity index, which is calculated from the S_{22} band, slightly increased its value upon low speed centrifugation, thus demonstrating the usefulness of ChNCs to readily stabilize SWCNTs in water. The dispersion in water and subsequent centrifugation of SWCNT/ChNC in moderately stronger conditions (at 13000 rpm for 4 min) nearly doubled the purity index of the SWCNTs, evidencing the potential of ChNCs to facilitate their purification by promoting their individualization through preferential adsorption and stabilization. These results are of the same order than those obtained with typical surfactants (e.g. SDBS)[8–10] and polymers (e.g. Pluronic®),[8,9] implying that a greener alternative to SWCNTs purification is possible via biopolymer nanocrystals without any detriment in their optical properties.

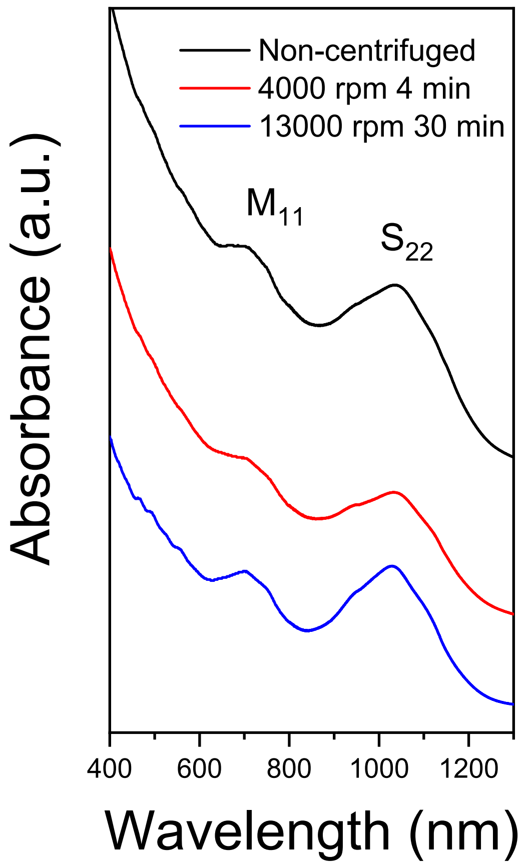

Figure S7. Absorbance results of SWCNT/ChNC inks at different centrifugation conditions in the visible and near infrared spectra.

Table S4. Purity index of SWCNT/ChNC inks at different centrifugation conditions

Sample	Area	Subtracted area	Purity index
Non-centrifuged	165.59	19.26	0.116
4000 rpm 4 min	108.31	13.10	0.121
13000 rpm 30 min	80.09	18.12	0.226

8. Homocoagulation analysis of SWCNT/ChNC and MWCNT/ChNC dispersions

We performed a homocoagulation analysis to further investigate the origin of the colloidal stability of the MWCNT/ChNC and SWCNT/ChNC inks. 1.5 mL of each dispersion was mixed with varying concentrations of NaCl (from 1 mol/L to 0.0001 mol/L), stored for 24 hours, and centrifuged at 4000 rpm for 4 minutes to induce the precipitation of the coagulated samples.

The photos (**Figure S8**) show the clear precipitation at NaCl concentrations above 0.1 mol/L for both compositions. At 0.01 mol/L, some degree of separation was also observed, though less pronounced. Below this concentration, the dispersions remained largely stable, showing minimal signs of coagulation, highlighting the high stability of the system and the affinity with the solvent. These findings suggest that the importance of the solvation forces helps to the colloidal stability of the dispersions, along with the electrostatic repulsion. The amphiphilic nature of the ChNCs could play a crucial role, with the hydrophobic face of the ChNCs adhering to the CNTs and the hydrophilic face solvated by water. This leads to the classification of these systems as lyophilic-like or hydrophilic-like colloids.

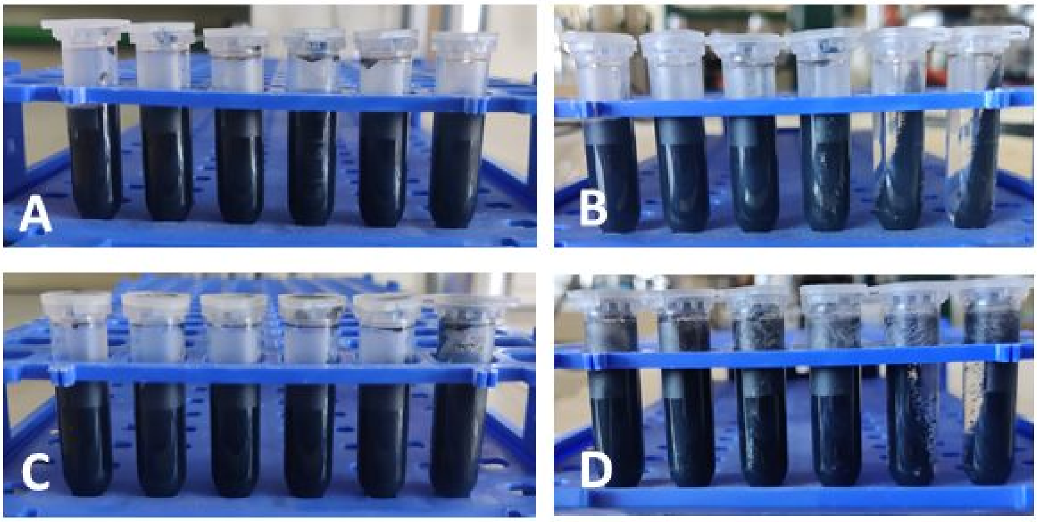

Figure S8. Homocoagulation analysis of SWCNT/ChNC (A and B) and MWCNT/ChNC (C and D) inks. Samples are arranged from left to right in order of increasing NaCl concentration (0, 10^{-4}, 10^{-3}, 10^{-2}, 0.01, 0.1 and 1 mol/L). Photographs A and C were taken immediately after NaCl addition, while B and D were taken after 24-hour storage and centrifugation at 4000 rpm for 4 minutes.

9. Sheet resistance results of the CNM/ChNC films

Once CNF/ChNC inks were spray coated, the resulting films displayed a somewhat lower electrical conductivity than those from SWCNTs or MWCNTs. The relatively higher R_S values could be explained according to two factors: their heterogeneity in size and a higher coverage of ChNCs arising from their intense interactions. This may reduce the number of electronic pathways and thus, the electrical properties of the film worsened respect to SWCNTs and MWCNTs.

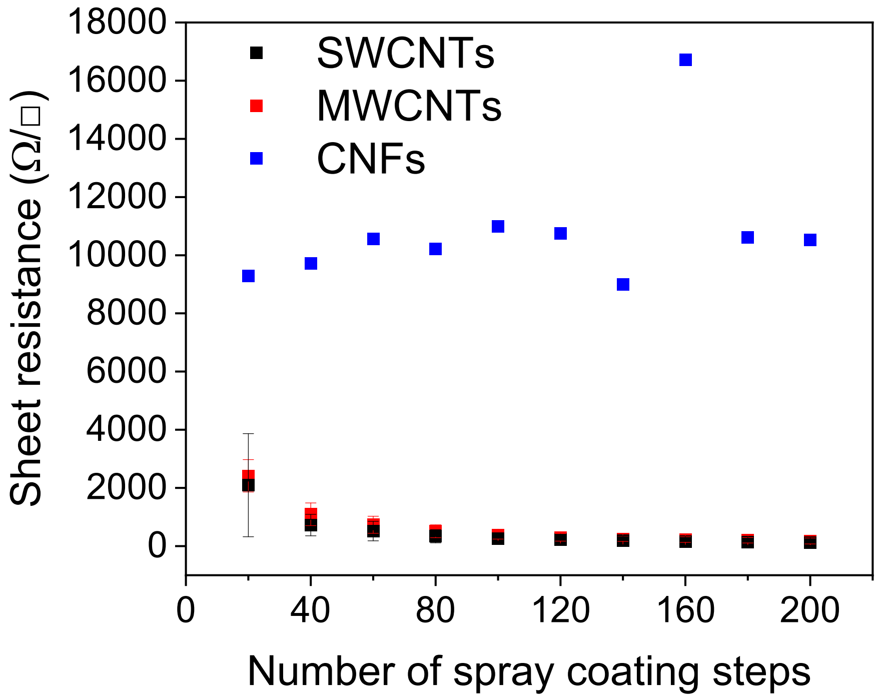

Figure S9. R_S of samples with increasing number of spray coating steps for SWCNTs (black squares), MWCNTs (red squares) and CNFs (blue squares) conductive films.

10. SEM images of the CNM/ChNC films

Figures S9A and S9C show that both films were uniform and densely packed, probably agglutinated by the chitin nanocrystals attached to the nanotubes. This is a characteristic aspect of the spray coated films. After the thermal treatment, it seems that the CNT bundles were more exposed than before (Fig. S9B and S9D). This supports that ChNCs had been pyrolyzed within the CNT matrix, thus agreeing with the decrease in thickness observed after the thermal treatment (Table 1).

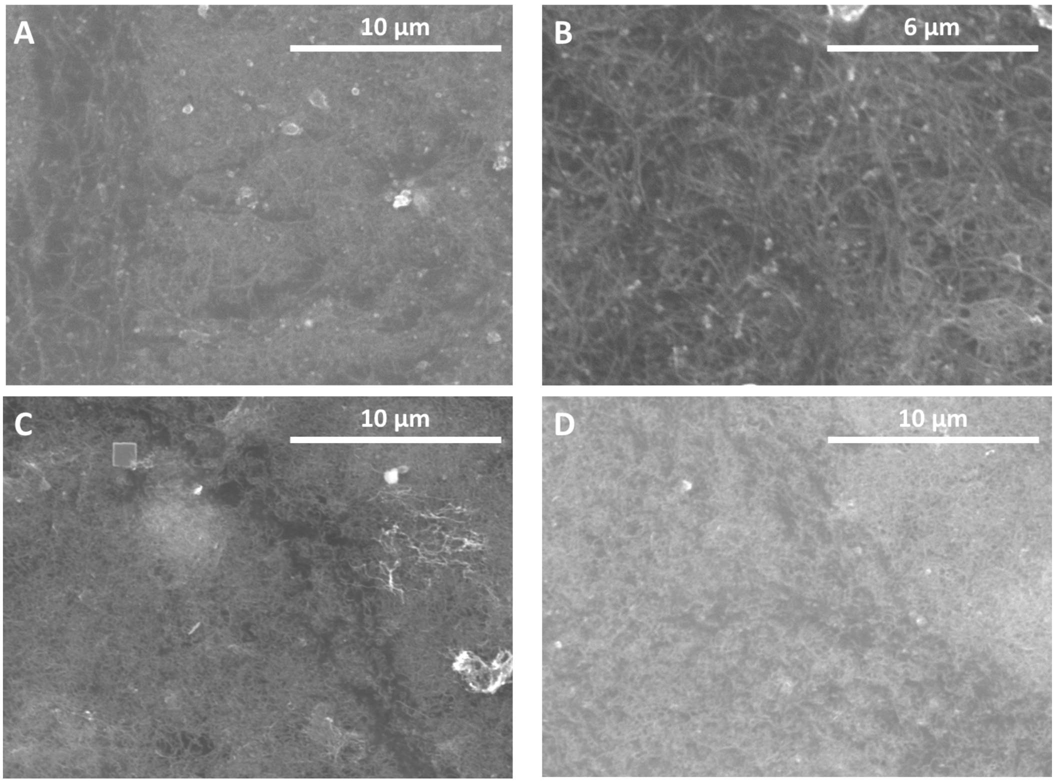

Figure S10. SEM before and after thermal treatment of CNM/ChNCs films: (A) non-treated SWCNT/ChNC, (B) treated SWCNT/ChNC, (C) non-treated MWCNT/ChNC and (D) treated MWCNT/ChNC.

11. Calculation of the CNM/ChNC films electrical conductivity

Electrical conductivity of the carbon-based conductive films was calculated according to **Equation S2**:

$$\sigma\ (S{\cdot}cm^{-1}) = \frac{1}{R_S(\Omega/\square){\cdot}t\ (cm)} \qquad \text{(S2)}$$

where R_S is the sheet resistance measured in Ω/□ and t is the thickness of the film, in cm.

12. XPS measurements of the CNM/ChNC films

The surface atomic composition of the CNM/ChNC films before and after the thermal treatment were ascertained by XPS (Figures S10 and S11 and table S5). In the first place, the as-prepared films show particular traits ascribable to a massive content in chitin, which agrees with the mass proportions of CNMs and chitin estimated by TGA and UV-vis spectroscopy. The main features observed are noticeable components coming from amide bonds (at ~289eV, ~401eV and ~532eV) and the residual amino groups (at ~399eV). The low content in sp^2 carbon (in the range of 5-12%, depending on the considered CNM) suggests that most of CNMs are heavily covered by ChNCs and are hardly visible on surface by the XPS technique. Then, after heating the films, there is a huge difference between the films before and after thermal treatment, and this is something particularly observed for carbon and nitrogen elements. In the first place, the C1s orbital drastically changes in shape and components, ending up in a graphitic-like shape, with a noticeable presence of C=C bonds seen at 284.4 eV (~50% for SWCNTs and ~35% for MWCNTs), and the disappearance of all features coming from chitin. The emergence of π-π* shakeup features (~291eV) also hints at an effective graphitization or aromatization of the sample as a consequence of the pyrolysis of ChNCs. The changes observed in the N1s region are also very revealing, since different C=N bonds can be observed, namely Pyrrolic nitrogen (at ~400eV, in MWCNTs) and pyridinic nitrogen (at ~398-399eV, in SWCNTs and MWCNTs). These new nitrogen species might be responsible of a change in the electrocatalytic effect of the treated films.

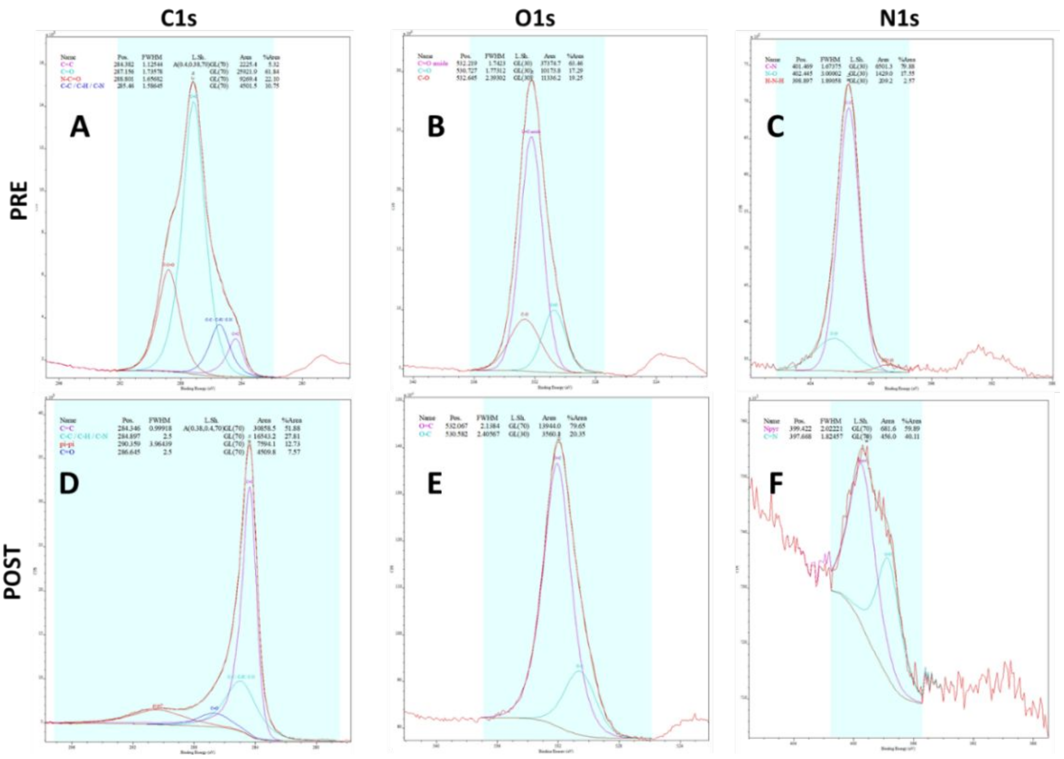

Figure S11. XPS results before (A-C) and after thermal treatment (D-F) of SWCNT/ChNC films separated in different orbitals: C1s (A and D), O1s (B and E) and N1s (C and F).

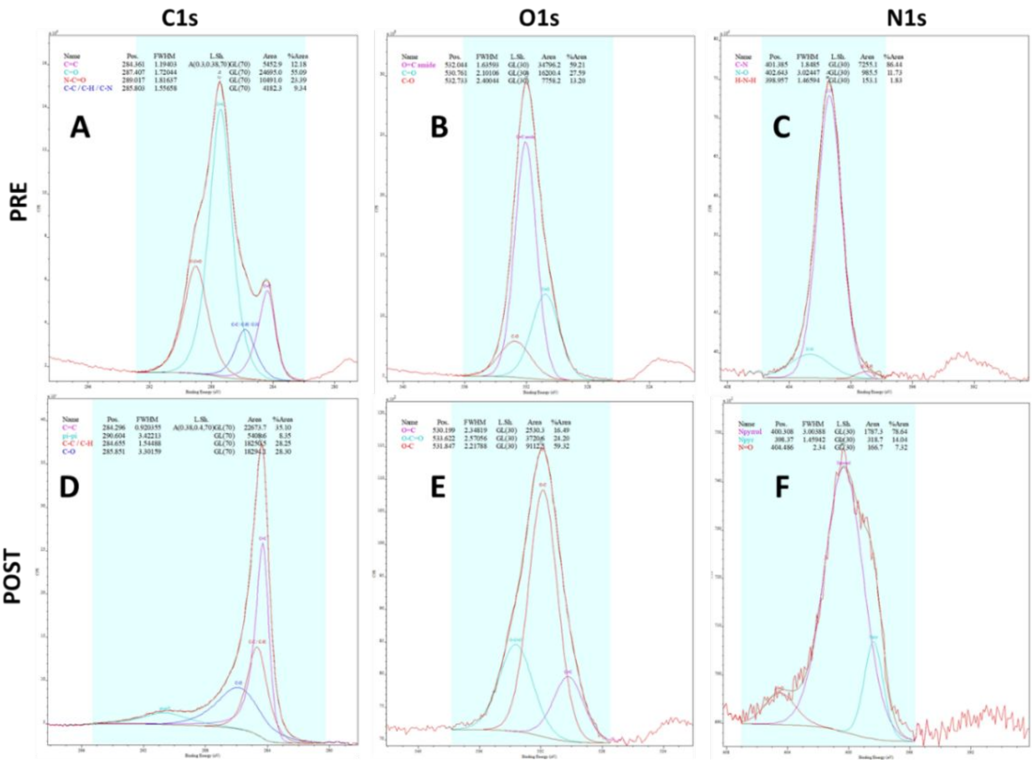

Figure S12. XPS results before (A-C) and after thermal treatment (D-F) of MWCNT/ChNC films focused in different orbitals: C1s (A and D), O1s (B and E) and N1s (C and F).

Table S5. Composition percentage of the different CNMs/ChNCs films before and after the thermal treatment determined by XPS.

Sample	%C	%O	%N	%S
SWCNT/ChNC non-treated	38.35	54.20	7.46	-
SWCNT/ChNC treated	70.22	19.39	7.78	2.61
MWCNT/ChNC non-treated	40.31	52.26	7.43	-
MWCNT/ChNC treated	77.6	17.25	3.26	1.89

13. Contact angle measurements of the CNM/ChNC films

Contact angle measurements were carried out evaluating the hydrophilicity of the films before and after thermal treatment. There is a substantial decrease in hydrophilicity in the pyrolyzed samples (Figure S10B, D and F) due to a higher exposure of CNMs.

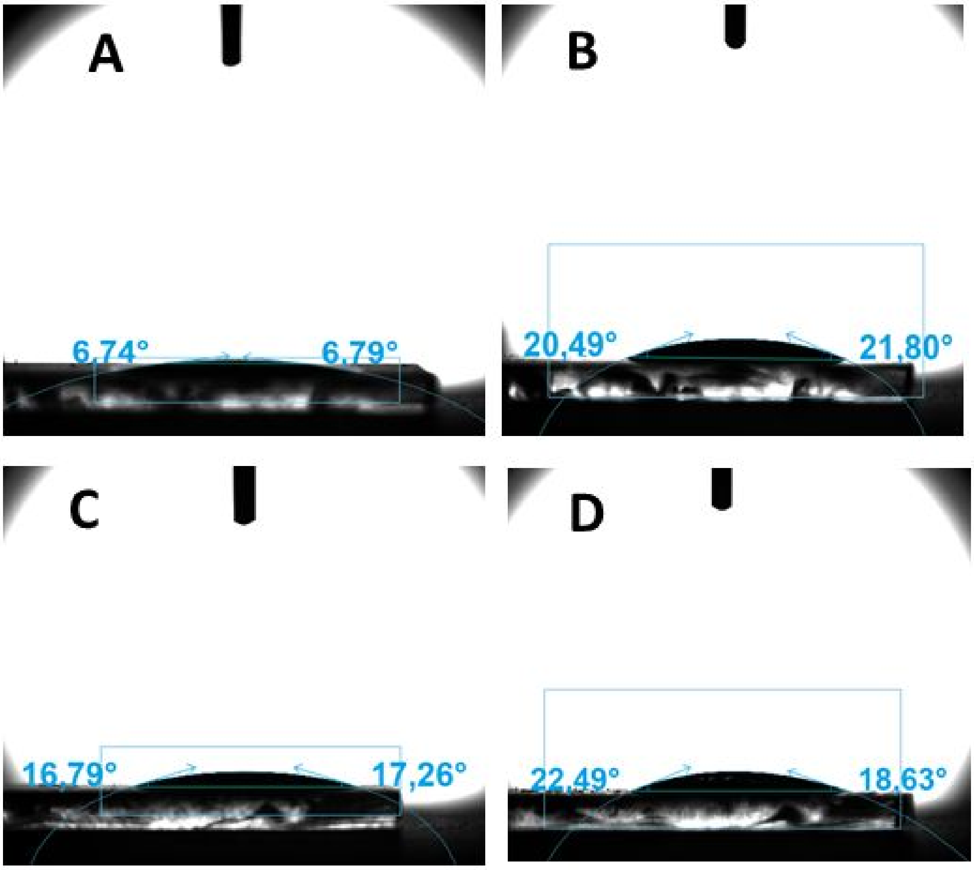

Figure S13. Contact angle measurements of CNM/ChNC films before and after the thermal treatment: (A) non-treated SWCNT/ChNC, (B) treated SWCNT/ChNC, (C) non-treated MWCNT/ChNC and (D) treated MWCNT/ChNC.

Table S5. Contact angle of water and the CNM/ChNC films

Sample	Contact angle (°) non-treated	Contact angle (°) treated
SWCNT/ChNC	6.3 ± 0.3	21.3 ± 0.3
MWCNT/ChNC	17.0 ± 0.2	20.6 ± 0.5

14. Calculation of the standard electrochemical rate constant

To study in detail the electrochemical properties of the different electrodes, the electron transfer rate constants from the peak separation have been determined from the CV experiments using an expanded numerical solution to Nicholson method.[11,12] The standard electrochemical rate constant (k^0) can be calculated from the cyclic voltammetry measurements assuming that the values of the anodic and cathodic charge transfer coefficients are α = 0.5 and equal diffusion coefficients (D) for the oxidized and reduced chemical entities. The calculations for obtaining the standard electrochemical rate constant were carried out following **Equation S3**:

$$k^0 = \sqrt{\left(\frac{\pi D n F \upsilon}{RT}\right)} \cdot \psi \qquad \text{(S3)}$$

Where:

n is the number of electrons that participate in the reaction (n = 1 for ferro/ferricyanide and n = 2 for hydroquinone)

F is the Faradaic constant (C/mol)

R is the gas constant (J/mol·K)

T is the temperature (K)

ψ is a dimensionless charge transfer parameter related to the peak separation[11]

υ is the employed scan rate during the cyclic voltammetry measurements?

D of the ferro/ferricyanide couple is $7 \cdot 10^{-6}$ (cm^2/s) and the D of the hydroquinone probe is $3 \cdot 10^{-6}$ (cm^2/s), being obtained from reference[13]

The employed ψ parameters were obtained from the expanded numerical solution of Nicholson's work.[11,12] In the case of ferro/ferricyanide this results in $6.309 \cdot 10^{-4}$ and $1.412 \cdot 10^{-2}$ for the non-treated and treated SWCNT/ChNC films. On the other hand, $5.248 \cdot 10^{-3}$ and $1.258 \cdot 10^{-1}$ were the values of the non-treated and treated MWCNT/ChNC films, respectively. Moreover, when hydroquinone is employed, only the pyrolyzed electrodes can be employed for such analysis due to the presence of redox peaks, something that the non-treated sample do not display. This results in $6.958 \cdot 10^{10}$ for SWCNT/ChNC and $2.6 \cdot 10^{-8}$ for MWCNT/ChNC electrodes.

On the other hand, the calculation of the k^0 for irreversible systems requires the use of a different equation and parameters. In particular, the Nicholson-Shain method was

followed for such purpose,[14] including a modification for the calculation of k^0 from the voltammetry (**Equation S4**) adapted at room temperature.[15]

$$k_0 = 1{,}11 \cdot D_0^{1/2} \cdot (E_p - E_{p/2})^{-1/2} \cdot \upsilon^{1/2} \qquad \text{(S4)}$$

Where:

D_0: diffusion coefficient of ascorbic acid ($5.8 \cdot 10^{-6}$ $cm^2 \cdot s^{-1}$) obtained from reference[13]

E_p: peak potential. The peak of N_2-treated SWCNTs/ChNCs electrode appears at 0.485 V (vs. Ag/AgCl), while in the case of the N_2-treated MWCNTs/ChNCs electrode is located at 0.284 V (vs. Ag/AgCl).

$E_{p/2}$: potential at which $I = I_p/2$. For the SWCNTs/ChNCs electrode, the value corresponds to 0.212 V (vs. Ag/AgCl), whereas the potential for the MWCNTs/ChNCs electrode is 0.071 V (vs. Ag/AgCl).

υ is the scan rate of the experiments.

15. References

(1) González-Domínguez, J. M.; Santidrián, A.; Criado, A.; Hadad, C.; Kalbáč, M.; Da Ros, T. Multipurpose Nature of Rapid Covalent Functionalization on Carbon Nanotubes. *Chemistry - A European Journal* **2015**, *21* (51), 18631–18641. https://doi.org/10.1002/chem.201503085.

(2) Setaro, A.; Adeli, M.; Glaeske, M.; Przyrembel, D.; Bisswanger, T.; Gordeev, G.; Maschietto, F.; Faghani, A.; Paulus, B.; Weinelt, M.; Arenal, R.; Haag, R.; Reich, S. Preserving π-Conjugation in Covalently Functionalized Carbon Nanotubes for Optoelectronic Applications. *Nat Commun* **2017**, *8*, 1–7. https://doi.org/10.1038/ncomms14281.

(3) Narkevicius, A.; Steiner, L. M.; Parker, R. M.; Ogawa, Y.; Frka-Petesic, B.; Vignolini, S. Controlling the Self-Assembly Behavior of Aqueous Chitin Nanocrystal Suspensions. *Biomacromolecules* **2019**, *20* (7), 2830–2838. https://doi.org/10.1021/acs.biomac.9b00589.

(4) Beck, S.; Bouchard, J.; Berry, R. Dispersibility in Water of Dried Nanocrystalline Cellulose. *Biomacromolecules* **2012**, *13* (5), 1486–1494. https://doi.org/10.1021/bm300191k.

(5) Zhang, Y.; Jiang, J.; Liu, L.; Zheng, K.; Yu, S.; Fan, Y. Preparation, Assessment, and Comparison of α-Chitin Nano-Fiber Films with Different Surface Charges. *Nanoscale Res Lett* **2015**, *10* (1). https://doi.org/10.1186/s11671-015-0926-z.

(6) Lertwattanaseri, T.; Ichikawa, N.; Mizoguchi, T.; Tanaka, Y.; Chirachanchai, S. Microwave Technique for Efficient Deacetylation of Chitin Nanowhiskers to a Chitosan Nanoscaffold. *Carbohydr Res* **2009**, *344* (3), 331–335. https://doi.org/10.1016/j.carres.2008.10.018.

(7) Yan, Y.; Ge, F.; Qin, Y.; Ruan, M.; Guo, Z.; He, C.; Wang, Z. Ultralight and Robust Aerogels Based on Nanochitin towards Water-Resistant Thermal Insulators. *Carbohydr Polym* **2020**, *248* (July), 116755. https://doi.org/10.1016/j.carbpol.2020.116755.

(8) Ansón-Casaos, A.; González-Domínguez, J. M.; Martínez, M. T. Separation of Single-Walled Carbon Nanotubes from Graphite by Centrifugation in a Surfactant or in Polymer Solutions. *Carbon N Y* **2010**, *48* (10), 2917–2924. https://doi.org/10.1016/j.carbon.2010.04.028.

(9) Ansón-Casaos, A.; González-Domínguez, J. M.; Lafragüeta, I.; Carrodeguas, J. A.; Martínez, M. T. Optical Absorption Response of Chemically Modified Single-Walled Carbon Nanotubes upon Ultracentrifugation in Various Dispersants. *Carbon N Y* **2014**, *66*, 105–118. https://doi.org/10.1016/j.carbon.2013.08.048.

(10) Ansón-Casaos, A.; González, M.; González-Domínguez, J. M.; Martínez, M. T. Influence of Air Oxidation on the Surfactant-Assisted Purification of Single-Walled Carbon Nanotubes. *Langmuir* **2011**, *27* (11), 7192–7198. https://doi.org/10.1021/la200730k.

(11) Nicholson, R. S. Theory and Application of Cyclic Voltammetry for Measurement of Electrode Reaction Kinetics. *Anal Chem* **1965**, *37* (11), 1351–1355. https://doi.org/10.1021/ac60230a016.

(12) Mahé, E.; Devilliers, D.; Comninellis, C. Electrochemical Reactivity at Graphitic Micro-Domains on Polycrystalline Boron Doped Diamond Thin-Films Electrodes. *Electrochim Acta* **2005**, *50* (11), 2263–2277. https://doi.org/10.1016/j.electacta.2004.10.060.

(13) Zoski; Cynthia G. *Handbook of Electrochemistry*; Elsevier, 2007.

(14) Nicholson, R. S.; Shain, I. Single Scan and Cyclic Methods Applied to Reversible, Irreversible, and Kinetic Systems. *Anal Chem* **1964**, *36* (4), 706. https://doi.org/10.1021/ac60210a007.

(15) González-Velasco, J. Determination of Standard Rate Constants for Electrochemical Irreversible Processes from Linear Sweep Voltammograms. *Electroanal* **1997**, 9 (11), 880. https://doi.org/10.1002/elan.1140091116.
